# Making Blank Faces Expressive: Chemical Approaches to the Modification of Chemically Inert Peptides

**DOI:** 10.1002/psc.70067

**Published:** 2026-01-30

**Authors:** Yoshitaka Moriyama, Hikari Sada, Takeshi Nanjo

**Affiliations:** ^1^ Graduate School of Pharmaceutical Sciences Kyoto University Kyoto Japan

**Keywords:** C–H functionalization, chemical modification, metal catalysis, peptide, radicals

## Abstract

Very minor structural modifications in peptides can result in significant changes in their function. To obtain analogues with such small structural but big functional differences compared to the original peptides, amino acid monomers are usually combined one‐by‐one from scratch, which is a simple and reliable but painfully laborious strategy. One alternative and very fascinating approach is the chemical modification of existing peptides, which allows for the rapid production of derivatives with fewer synthetic steps. However, such approaches generally target the reactive functional groups in cysteine and lysine residues, particularly in the case of larger peptides, and the modification of peptides that do not feature these functionalities is more difficult. Nevertheless, chemists have also been exploring methods that can be applied even to such chemically inert peptides based on recent advances in C–H activation and hydrogen atom transfer (HAT) chemistry. If successful, these strategies would represent a breakthrough in terms of obtaining unusual peptide structures in a time‐ and cost‐effective manner. This review focuses on recent attempts to achieve such ambitious chemical modifications, albeit that these are currently limited to relatively small peptides.

## Graphical abstract

As an alternative to the conventional approach, which combines amino acid monomers in a one‐by‐one fashion to peptide derivatives, the chemical modification of existing peptides has attracted significant attention in recent years. However, such approaches generally target the reactive functional groups in cysteine and lysine residues, particularly in the case of larger peptides, and the modification of peptides that do not feature these functionalities is more difficult. This review focuses on recent attempts to achieve such more ambitious chemical modifications.

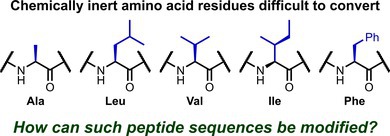

## 1 Introduction

**FIGURE 1:**
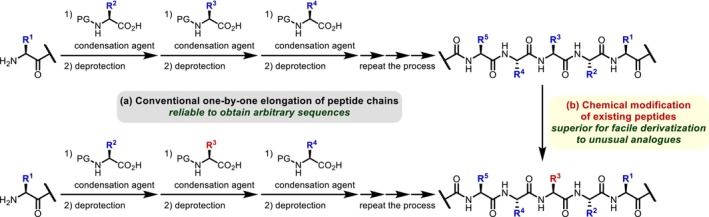
Chemical modification of peptides.

Peptides form a class of biomolecules generated from the dehydrative condensation of amino acids. In addition to the innumerable peptides derived from the 20 proteinogenic amino acids, which exhibit a variety of functions in living organisms, artificial peptides such as macrocyclic peptides and stapled peptides have been attracting attention as a new modality in midsize‐molecule drug discovery [1, 2]. Thus, the need for synthetic methods that readily provide access to such unique unnatural structures is becoming increasingly urgent. The chemical synthesis of peptides is typically achieved via the dehydrative condensation of amino acids in a one‐by‐one fashion (Figure 1a). Specifically, Fmoc solid‐phase synthesis is a well‐established method that readily allows synthesizing even long‐chain peptides with arbitrary sequences [3, 4, 5]. As an alternative to this viable but linear synthetic route to peptide derivatives, the chemical modification of existing peptides has attracted significant attention in recent years (Figure 1b). This approach is very effective, especially in terms of (1) allowing later modification of the function of peptides, (2) preparing motifs that are difficult to introduce via the linear condensation approach, and (3) obtaining analogues of existing peptides. To date, various transformations that have initially been reported for simple nonpeptide small molecules have been expanded to peptides. However, most current practical methods target reactive functional groups on peptide side chains (e.g., cysteine [Cys], lysine [Lys], and tyrosine [Tyr]), which limits the scope of situations and reaction sites to which they can be applied; reactions of “inert peptide sequences” that do not contain these functional groups still remain difficult [6].

In this review, we will cover strategies and reactions other than the conventional functional group transformation (FGT) of reactive side chains that have been developed for converting peptides into their analogues, that is, the conversion of C–H bonds in the side or main chains of peptides, which are usually considered to be unreactive sites. The review does not cover the transformation of amino acid monomers; only reactions of so‐called peptides, in which two or more amino acids are connected through peptide bonds, are discussed. In addition, strategies based on the elongation or cleavage of the peptide chain are also excluded, as the purpose of this review is to introduce emerging approaches to synthesize analogues of existing peptides. For enzymatic transformations, the reader is directed to an appropriate review dedicated to such reactions [7].

## 2 Conversion of Inert C–H Bonds in Peptide Side Chains

**FIGURE 2:**
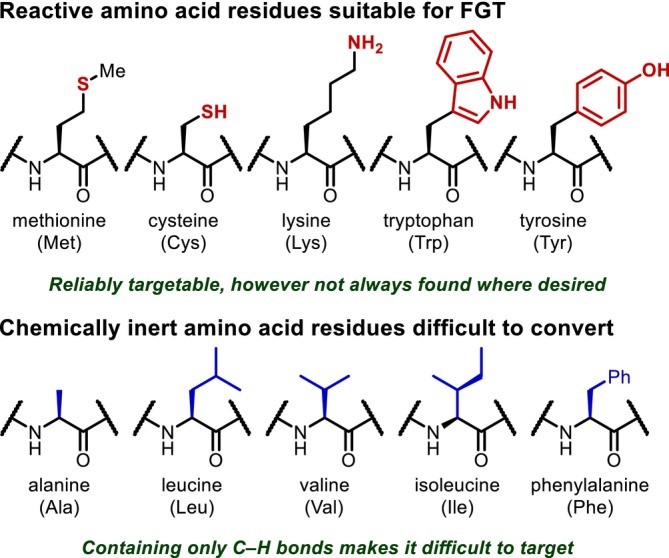
Chemical modification of peptide side chains.

Side chains are the most effective site for adding diversity to peptide structures, and transformations that can supply a wide variety of analogues of an existing peptide structure are extremely important. However, not all peptide side chains contain functional groups suitable for conventional FGT, which severely limits the situations in which the derivatization approach is effective (Figure 2). In cases where the peptide side chain does not feature a reactive functional group, the only option is the conversion of a C(sp^3^)–H bond, which is generally difficult in synthetic organic chemistry. Methodologies to convert C–H bonds have been intensively explored in recent years, as such transformations enable the rapid construction of complex structures via the conversion of ubiquitous C(sp^3^)–H bonds into various carbon skeletons or functional groups. Many such methods have been developed for nonpeptide molecules, and their applications to peptides gradually emerge.

The reactions currently applied to peptides can be roughly divided into three categories: (1) the nondirected transformation of remote C(sp^3^)–H bonds promoted by highly electrophilic species, (2) radical‐mediated intramolecular hydrogen atom transfer (HAT) processes, and (3) directed, transition metal‐catalyzed C–H activation reactions. Each of these categories will be discussed in its own section (*vide infra*). In addition, the transformation of aromatic C(sp^2^)–H bonds in aromatic amino acids such as phenylalanine (Phe) remains particularly difficult, and thus, we will introduce recent developments in this area as well.

### 2.1 Nondirected Transformation of Remote C(sp^3^)–H Bonds Promoted by Electrophilic Reagents

**FIGURE 3:**
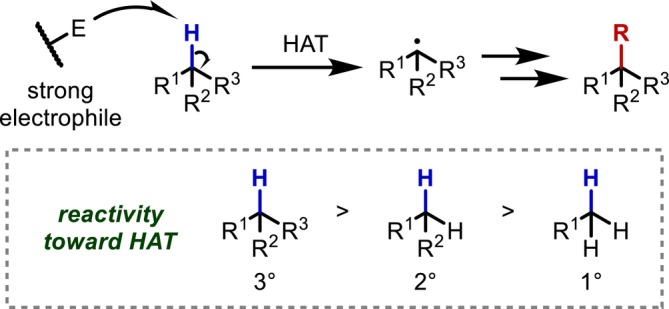
Strong electrophile‐mediated HAT in aliphatic moieties.

The most conventional approach to the reaction of aliphatic C(sp^3^)–H bonds is radical‐mediated HAT. Strong electrophiles can react with the C(sp^3^)–H bonds of typically inert aliphatic chains to enable various transformations using the generated alkyl radicals; in particular, hydrogen abstraction promoted by highly electrophilic radicals such as hydroxy, alkoxy, and halogen radicals is well established (Figure 3). A major advantage of this reaction is that, unlike other approaches described later, it does not require directing groups in the substrate. Furthermore, the HAT reaction favors electron‐rich C(sp^3^)–H bonds that generate stable alkyl radicals upon reaction, meaning that tertiary C–H bonds, which are too bulky to be converted via transition metal‐mediated catalysis, preferentially undergo this reaction.

However, several challenges arise in the application of this methodology to peptide chains. First, most C(sp^3^)–H bonds in proteinogenic peptide side chains are deactivated by the inductive effect of the electronegative main chain, and few aliphatic C–H bonds exhibit good reactivity toward HAT reactions. Generally, the introduction of an electronegative atom or an electron‐withdrawing substituent decreases the electron density of aliphatic chains at the α–γ positions due to the inductive effect. The HAT reaction is inherently difficult due to the strength of the C–H bond, and thus, even minor electronic effects have a significant impact. Additionally, the scarcity of tertiary C–H bonds in peptide side chains further exacerbates reactivity issues. The second issue is that strong oxidants capable of inducing HAT reactions in the aliphatic chains often cause oxidative side reactions. This limits the scope of compatible functional groups in the side chains. Moreover, strong reaction conditions often affect even the amide groups of the peptide main chain, making it impossible to achieve sufficient reactivity to convert less reactive C(sp^3^)–H bonds in peptide side chains. Due to the tradeoff between reactivity and chemoselectivity, very few methods have been demonstrated to be applicable to a wide range of peptide compounds.

**FIGURE 4:**
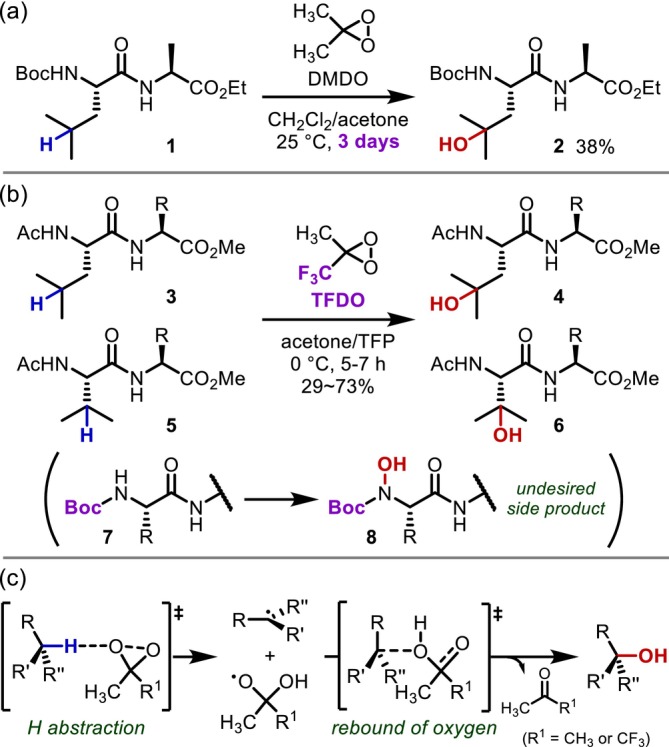
Dioxirane‐mediated hydroxylation of tertiary C(sp^3^)–H bonds; (a) with DMDO; (b) with TFDO. (c) A plausible reaction mechanism.

In 1997, Mincione and Saladino achieved the first oxidative transformation of C(sp^3^)–H bonds in peptide side chains (Figure 4a) [8]. They demonstrated the hydroxylation of tertiary C–H bonds at the γ‐position of leucine (Leu) residues by treating dipeptides with dimethyl dioxirane (DMDO) [8, 9], a powerful oxygen‐transfer reagent [10] long known to oxidize C(sp^3^)–H bonds [11]. However, the applicable reaction sites are limited to the tertiary γ‐C–H bonds of Leu, and the conversion is usually < 50%, even after prolonged reaction times (typically days). Subsequently, in 2007, Williard and co‐workers reported that the oxidation of dipeptides or tripeptides using methyl (trifluoromethyl)dioxirane (TFDO) [12, 13], which exhibits much stronger activity than DMDO, makes the reaction proceed within a few hours for the tertiary C–H bonds of Leu or valine (Val) residues (Figure 4b) [14, 15]. However, *N*‐hydroxylation of the carbamate competes with the desired reaction, preventing the use of the *t*‐butoxycarbonyl (Boc) protecting group, and the yield of the desired oxidation product remained low to moderate. The C–H oxidation using dioxirane was initially thought to proceed via an insertion mechanism; however, recent computational studies have suggested that a radical mechanism, in which the formation of radical pairs followed by the rapid rebound of the oxygen, is more likely (Figure 4c) [16, 17].

**FIGURE 5:**
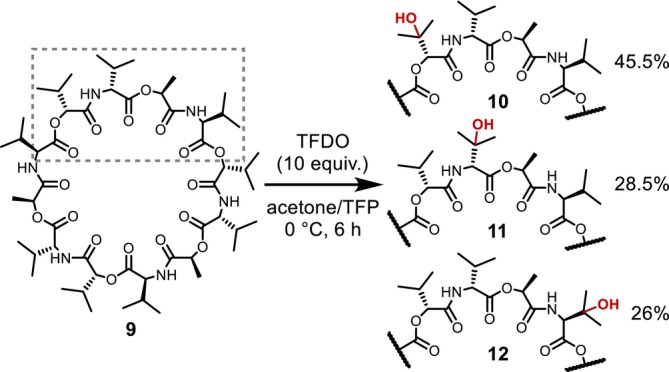
Hydroxylation of the Val residue in valinomycin.

In 2011, Annese and Williard reported the application of C–H oxidation using TFDO to the β‐hydroxylation of the Val side chain of valinomycin (**9**), a macrocyclic depsipeptide antibiotic, demonstrating that this reaction is an attractive approach for the chemical postmodification of complex or large peptide molecules, despite some remaining challenges in terms of reactivity and functional group compatibility (Figure 5) [18].

**FIGURE 6:**
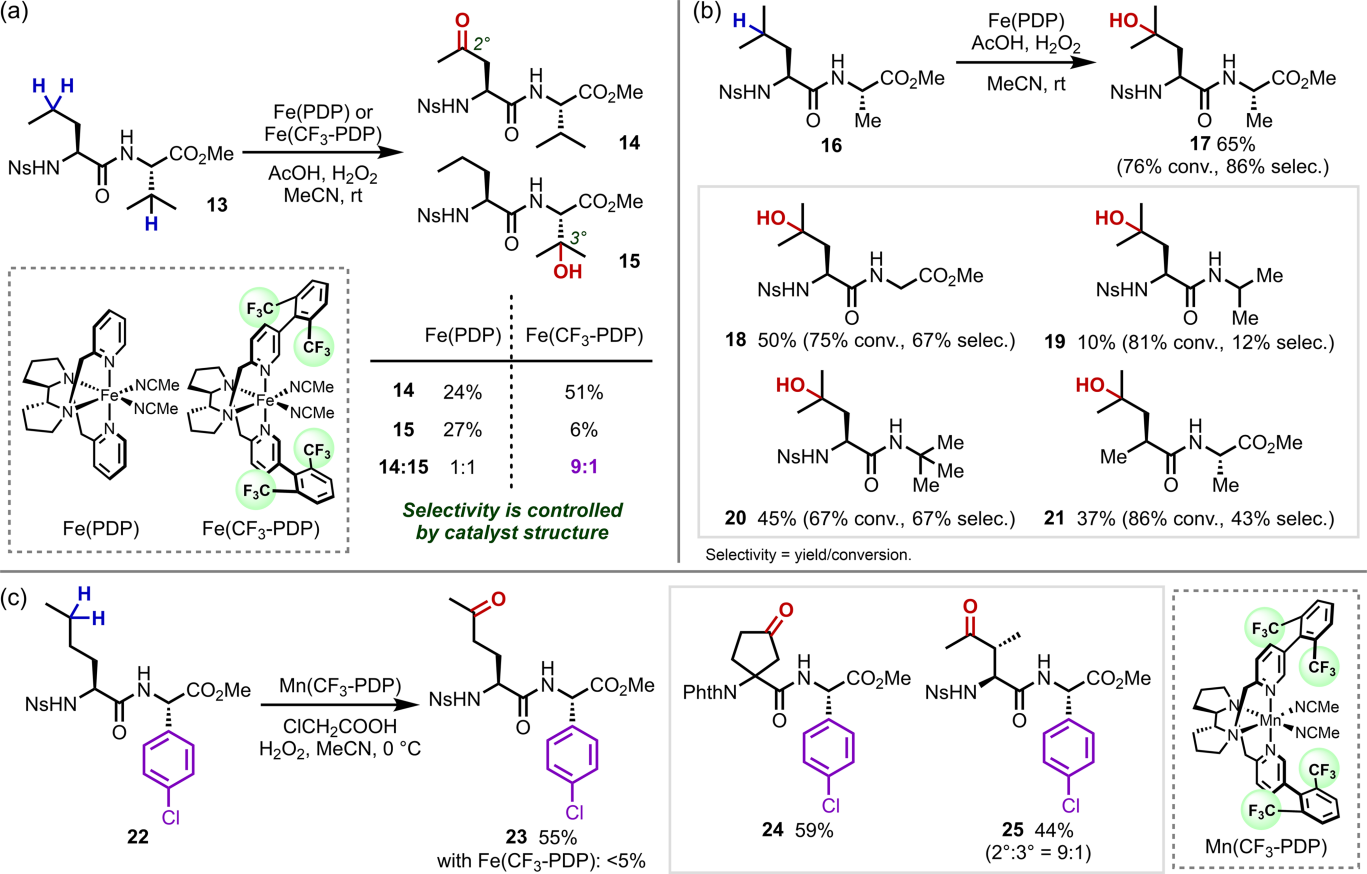
Predictable, site‐selective, and divergent C(sp^3^)–H oxidation of peptides enabled by small‐molecule Fe or Mn catalysts. (a) Catalyst control over the site‐selectivity. (b) Importance of the peptide backbone for the chemoselectivity. (c) Mn(CF_3_‐PDP) for the C(sp^3^)–H oxidation of aromatic compounds.

After these pioneering studies using dioxirane, no further reports on the HAT‐mediated transformation of C(sp^3^)–H bonds in peptides emerged for some time due to the lack of appropriate reactions. In 2007, Chen and White [19, 20] developed the groundbreaking Fe(PDP) catalyst for aliphatic C–H oxidation and demonstrated that its site‐selectivity could be predicted by considering electronic, steric, and stereoelectronic effects. In 2013, the same group developed the Fe(CF_3_‐PDP) catalyst, which is highly sensitive to steric effects and can react selectively with secondary C–H bonds in the presence of tertiary C–H bonds, demonstrating for the first time that the site‐selectivity of the transformation of aliphatic C(sp^3^)–H bonds can be changed via catalyst control rather than substrate control using a small molecular catalyst [21]. This group not only achieved the C(sp^3^)–H oxidation of chemically inert aliphatic side chains in dipeptides but also succeeded in selectively oxidizing the tertiary C–H bond at the β‐position of a Val residue and the secondary C–H bond at the γ‐position of a norvaline (Nva) residue by using either Fe(PDP) or Fe(CF_3_‐PDP) as the catalyst (Figure 6a). These small‐molecule Fe catalysts are also effective for the chemical modification of peptides initiated by oxidation of the N‐α position of proline (Pro) residues (*vide infra*) and have proven useful for the C–H oxidation of a wide range of amino acids and peptides [22]. Later, it was found that simple amides are not tolerated using these Fe catalysts, and it became clear that the reason that the C–H oxidation proceeded so well in peptides was that the amides in the peptide backbone were electronically and sterically shielded by surrounding electron‐withdrawing substituents (Figure 6b) [23]. In 2019, the same group developed Mn(CF_3_‐PDP) [24], a catalyst effective for the C–H oxidation of aromatic compounds; in contrast to Fe catalysts, which readily oxidize aromatic rings, this Mn catalyst can selectively oxidize secondary C–H bonds in peptides that contain phenylglycine derivatives (Figure 6c).

**FIGURE 7:**
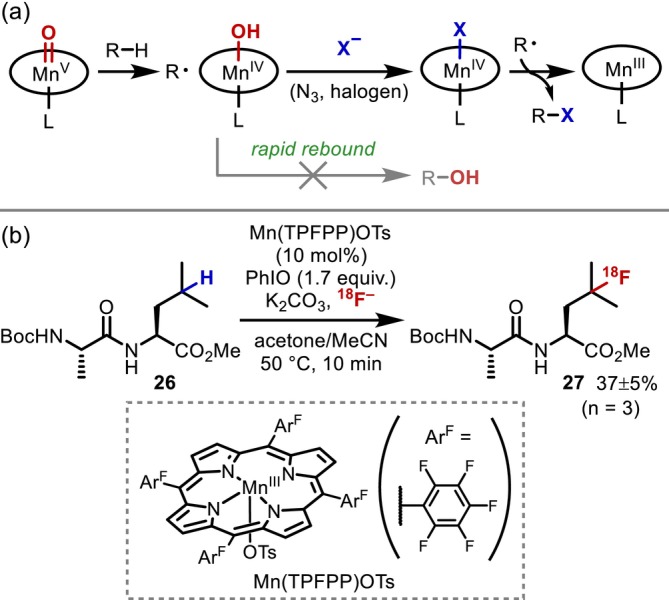
Mn‐porphyrin‐catalyzed aliphatic C(sp^3^)–H functionalization. (a) Mechanistic scenario. (b) Installation of ^18^F into Leu residues.

Relatedly, the group of Groves has intensively studied C(sp^3^)–H functionalization reactions using metalloporphyrin complexes, inspired by the enzymatic C–H oxidation catalyzed by the heme‐thiolate monooxygenase cytochrome P450 [25, 26]. In 1979, they reported the Fe‐porphyrin‐catalyzed C–H oxidation of alkanes [27], before they moved to the Mn analogue, which exhibits higher reactivity than the Fe complex [28]. A key advantage of this Mn‐porphyrin system is its applicability to other functionalizations, including halogenation and azidation, in addition to hydroxylation [29, 30, 31], despite the fact that this catalyst was initially considered to not be applicable to such transformations due to the rapid oxygen rebound (Figure 7a). This method has since been applied to various molecules, that is, mainly hydrocarbons including terpenes. Surprisingly, its use in peptides was not reported until 2018, when successful ^18^F labeling of a Leu residue in a dipeptide **26** was achieved (Figure 7b) [32].

**FIGURE 8:**
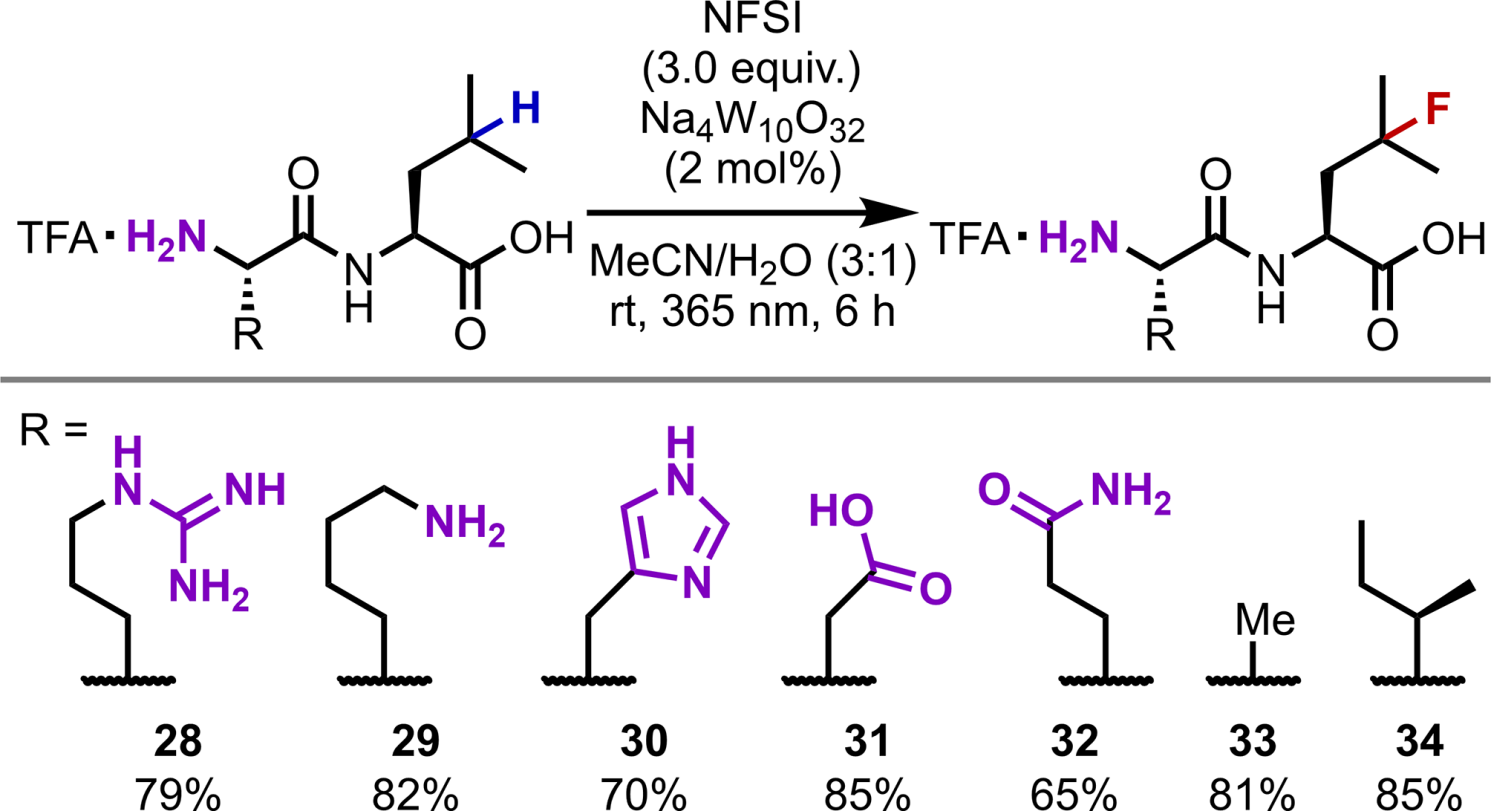
γ‐C(sp^3^)–H Fluorination of Leu residues in unprotected peptides promoted by the photoirradiation of decatungstate.

Another notable relevant study is the photocatalytic reaction using polyoxometalate reported by Combs‐Walker and Hill [33]. The authors found that the decatungstate anion exhibits remarkably high HAT activity toward aliphatic C(sp^3^)–H bonds under ultraviolet (UV) irradiation. This catalyst has since been applied to various reactions including C–C bond formation via the addition of radical‐trapping agents [34, 35]. Interestingly, despite its high reactivity, high chemoselectivity is maintained, and in 2014, Britton demonstrated the C(sp^3^)–H fluorination of amino acids with unprotected N‐termini [36]. Furthermore, in 2018, the γ‐selective C(sp^3^)–H fluorination of Leu residues in peptides wherein both the main chain and side chain functionalities were unprotected was achieved by the same group (Figure 8) [37]. This method has since been applied to hexapeptides, which are relatively large molecules in terms of HAT chemistry, where it exhibits excellent functional group compatibility with the exception of some electron‐rich aromatic rings and sulfur‐based functional groups. Noteworthily, the introduction of ^18^F, which is useful for positron emission tomography (PET) imaging, has been successfully achieved via this strategy.

**FIGURE 9:**
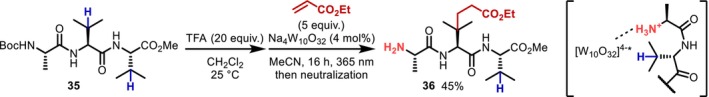
Electrostatic interaction‐promoted site‐selective β‐C–H functionalization of Val residues.

Torigoe and Kuninobu have achieved the conversion of tertiary C–H bonds to C–C bonds at the β‐position of Val residues in dipeptides and tripeptides using electron‐deficient alkenes as radical acceptors; here, the amino group on the N‐terminal was also left unprotected [38]. The authors suggested that the reaction at the N‐terminal side was promoted for peptides that contain multiple valine residues due to the effect of the electrostatic proximity between the ammonium cation and the decatungstate anion (Figure 9).

**FIGURE 10:**
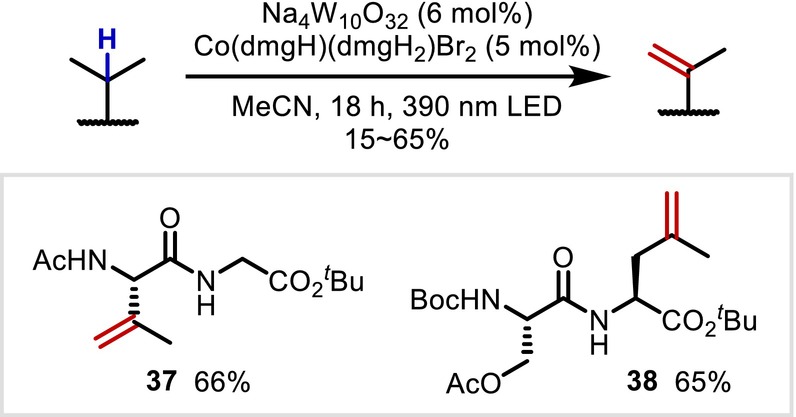
Co/W cocatalyzed desaturation of peptide side chains.

In 2024, Wendlandt reported the desaturation of peptide side chains promoted by a Co/W cocatalyst system [39]. The authors proposed that alkyl radicals generated through HAT induced by an excited decatungstate react with the Co catalyst to provide thermodynamically unstable terminal olefins via a metal‐hydride HAT (MHAT) mechanism. This method was demonstrated to be applicable to a wide range of peptide compounds, similar to other decatungstate‐catalyzed reactions (Figure 10).

**FIGURE 11:**
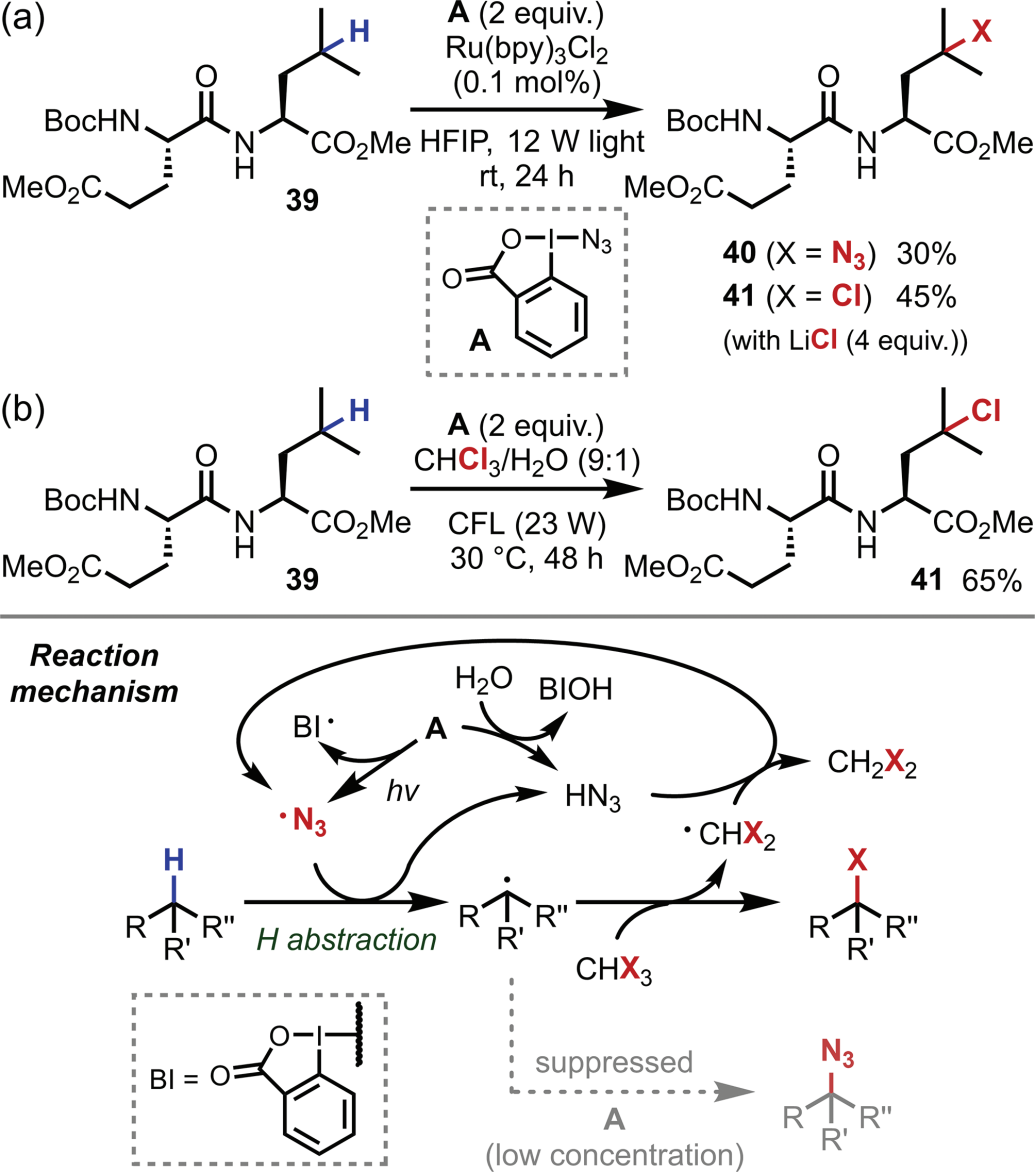
Zhdankin reagent‐mediated γ‐C–H functionalization of Leu residues in dipeptides. (a) Azidation and chlorination of tertiary C–H bonds. (b) Chlorination with chloroform as the chlorine source.

Numerous reactions in which nonmetallic electrophiles are involved in HAT have also been reported, with hypervalent iodine (III) reagents [40, 41] being representative examples. In 2016, Chen reported aliphatic C(sp^3^)–H functionalization using the Zhdankin reagent (**A**) [42], a relatively stable and easy‐to‐handle cyclic hypervalent iodine reagent [43]. The C(sp^3^)–H azidation is promoted by the Zhdankin reagent **A** in the presence of Ru (bpy)_3_Cl_2_ under visible light irradiation, and the addition of chloride and bromide ions results in chlorination and bromination, respectively. The same authors also applied these transformations to dipeptides to convert the tertiary C–H bond at the γ‐position of the Leu residue (Figure 11a). Subsequently, modified protocols for chlorinations using the Zhdankin reagent were reported; in 2021, Hartwig reported conditions employing stoichiometric amounts of Cu/bipyridine instead of light irradiation [44], whereas in 2023, Chen and Wang reported photoreaction conditions without any photo‐ or metal‐based catalysts using haloform as a halogen bonding donor (Figure 11b) [45]. However, these studies reported only one example of a peptide (**39**) each, both of which involved the conversion of a tertiary C–H bond at the γ‐position of the Leu residue. It should also be noted here that an azide moiety on the hypervalent iodine (III) is essential for these reactions, irrespective of whether the azide is incorporated into the final product; this is likely due to an azide radical generated by light irradiation of the hypervalent iodine species, which is responsible for the hydrogen abstraction step [43, 45].

**FIGURE 12:**
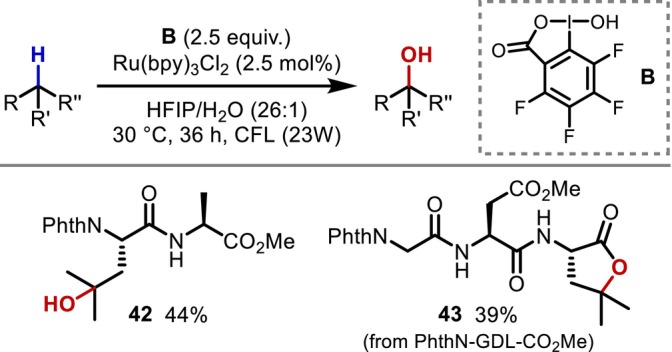
Perfluorinated benziodoxole‐mediated azide‐free γ‐C–H functionalization.

In 2017, Liu and Chen developed azide‐free C(sp^3^)–H oxidation reaction conditions using perfluorinated benziodoxole (**B**) and applied it to the hydroxylation of the γ‐C–H bonds of Leu in a dipeptide (**42**) and a tripeptide (**43**) [46]. The authors proposed that the generated carboxyl radicals, which have enhanced electrophilicity, serve as hydrogen abstraction agents rather than azide radicals (Figure 12).

**FIGURE 13:**
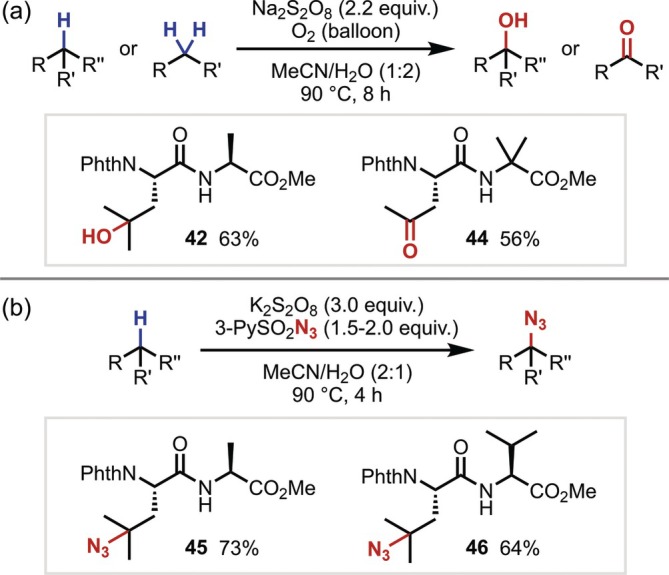
(a) Peroxodisulfate‐mediated oxidation of aliphatic C–H bonds in dipeptides. (b) Azidation with 3‐pyridinesulfonyl azide.

In 2016, aliphatic C–H oxidation using sodium peroxodisulfate (Na_2_S_2_O_8_) was reported. Gao and Shi demonstrated the oxidation of relatively unreactive C(sp^3^)–H bonds, even in the vicinity of electron‐withdrawing groups, by heating Na_2_S_2_O_8_ in an aqueous solvent under an oxygen atmosphere, thereby achieving secondary and tertiary C(sp^3^)–H oxidation in dipeptides (Figure 13a) [47]. Subsequently, Li and Shi [48] also succeeded in the azidation of the γ‐position of Leu in dipeptides by conducting the same reaction in the presence of sulfonyl azide rather than under an oxygen atmosphere (Figure 13b).

**FIGURE 14:**
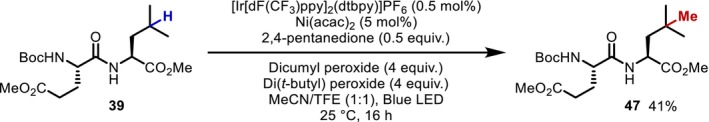
Selective methylation of tertiary C–H bonds promoted by Ir/Ni cocatalysis.

In 2024, Hartwig reported C–H methylation based on C–H abstraction by alkoxy radicals [49]. Here, Ir photocatalysis generates alkoxy radicals and methyl radicals from dicumyl peroxide; the alkoxy radicals mediate C–H abstraction, whereas the methyl radicals act as a methyl source via Ni catalysis, leading to the selective methylation of tertiary C–H bonds. Although only one example of a peptide (**39**) substrate was reported, this approach was also applied to the γ‐C–H methylation at the Leu residue in a dipeptide (Figure 14).

### 2.2 Radical‐Mediated Intramolecular Hydrogen Atom Transfer (HAT) Processes

Hydrogen abstraction promoted by an electrophilic radical within the same molecule is a useful strategy to effectively generate alkyl radicals at specific positions. Such intramolecular HAT reactions triggered by radical generation from *N*‐haloamines are commonly known as the Hofmann–Löffler–Freytag reaction [50, 51]. Among the intramolecular HAT processes, 1,5‐HAT, which passes through a six‐membered transition state, is particularly advantageous, and unless there are special conformational concerns, the reaction proceeds exclusively at the δ‐position relative to the atom where the radical is generated. Even aliphatic C(sp^3^)–H bonds in peptide side chains, which exhibit low reactivity toward intermolecular HAT as described in Section 2.1, can be effectively functionalized using the 1,5‐HAT approach, taking advantage of this intramolecular reaction. On the other hand, unlike intermolecular HAT, this approach requires the preinstallation of a radical precursor motif into the peptide. Moreover, the fact that the introduction site is limited to the N‐terminal amino group or C‐terminal carboxyl group of the peptides (except in the case of C(sp^3^)–H chlorination using the “*N*‐chloropeptide strategy”; for details, see Section 5) significantly reduces the potential of this approach.

**FIGURE 15:**
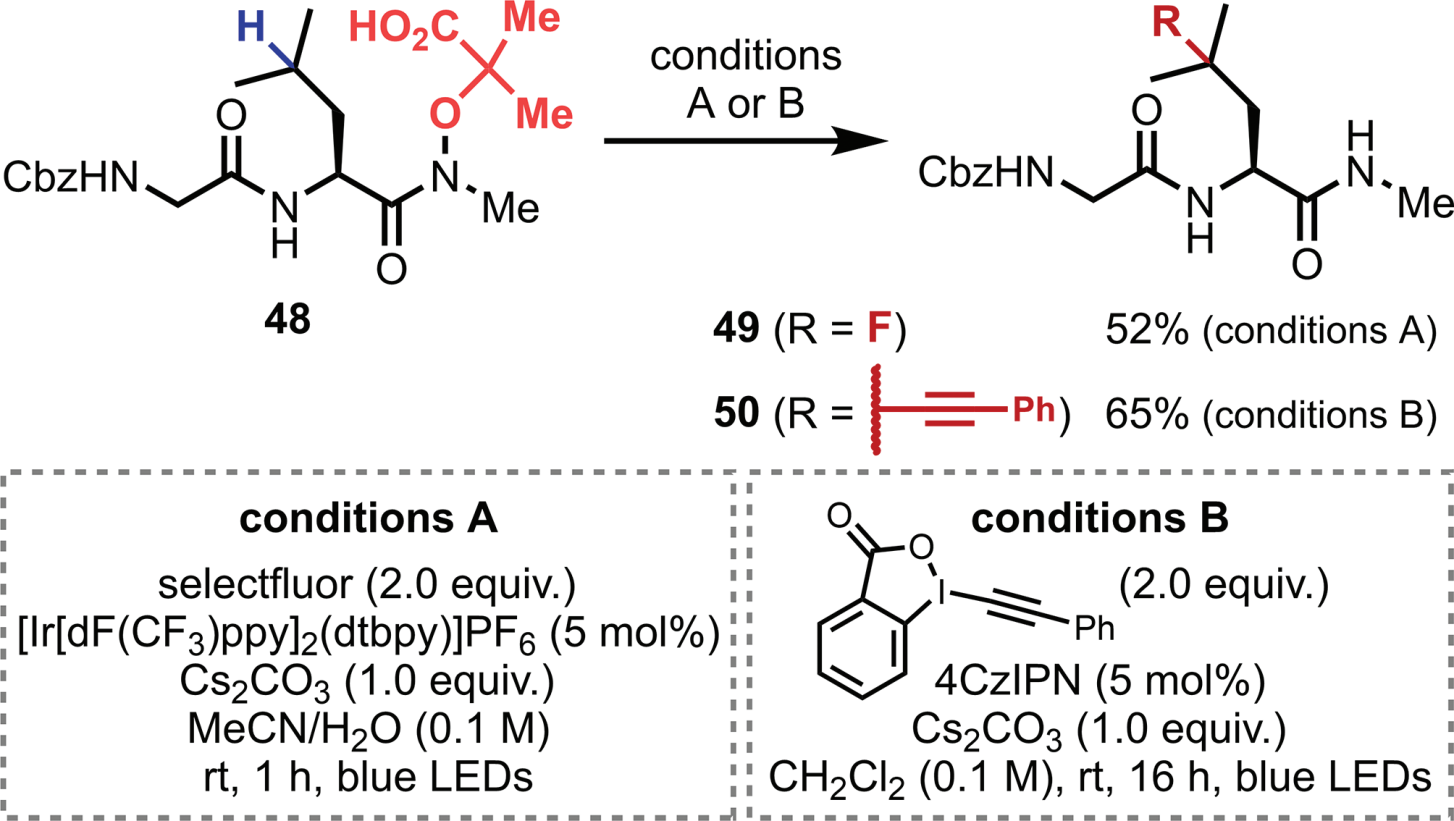
1,5‐HAT‐mediated fluorination and alkynylation of γ‐C(sp^3^)–H bonds of the Leu residue at the C‐terminal of a dipeptide.

Probably due to these issues, the first report on the side‐chain modification of peptides based on 1,5‐HAT did not appear until 2018, when Leonori and co‐workers achieved the photocatalytic 1,5‐HAT reaction and applied the strategy to the γ‐C(sp^3^)–H fluorination and alkynylation of the Leu residue at the C‐terminal of a dipeptide (**48**) by introducing a special hydroxamate derivative on the C‐terminus (Figure 15) [52]. In this reaction, the carboxy group undergoes single‐electron oxidation with the assistance of an excited photocatalyst, which is followed by decarboxylation and β‐scission to release acetone and generate an amidyl radical that exhibits high reactivity toward 1,5‐HAT.

**FIGURE 16:**
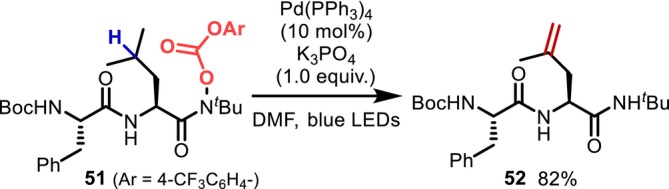
1,5‐HAT‐mediated desaturation of a Leu residue at the C‐terminal of a dipeptide.

In 2021, Jin and Yu [53] achieved the palladium‐catalyzed desaturation of Leu residues in a dipeptide containing *O*‐acyl hydroxamic acid at the C‐terminal (Figure 16). Initially, a photoexcited zero‐valent palladium induces the single electron reduction of *O*‐acyl hydroxamic acid, followed by the formation of an amidyl radical, which undergoes 1,5‐HAT, recombination of the alkyl radical catalyzed by palladium, and β‐hydride elimination to generate a terminal alkene.

**FIGURE 17:**
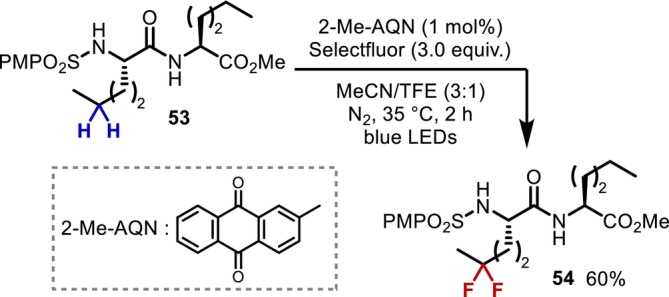
1,5‐HAT‐mediated difluorination of Nle residues at the N‐terminal of dipeptides.

In 2023, Li reported the difluorination of δ‐C(sp^3^)–H bonds in norleucine (Nle) residues in peptide chains (Figure 17) [54]. The sulfonamidyl radical generated by the single electron oxidation and subsequent deprotonation of the *p*‐methoxysulfonamide group introduced at the N‐terminal is responsible for intramolecular hydrogen abstraction. This reaction is the only known example in which a nonactivated secondary C(sp^3^)–H bond in a peptide is converted through an intramolecular HAT process, except for the C(sp^3^)–H chlorination achieved by the *N*‐chloropeptide strategy described in Section 5.

**FIGURE 18:**
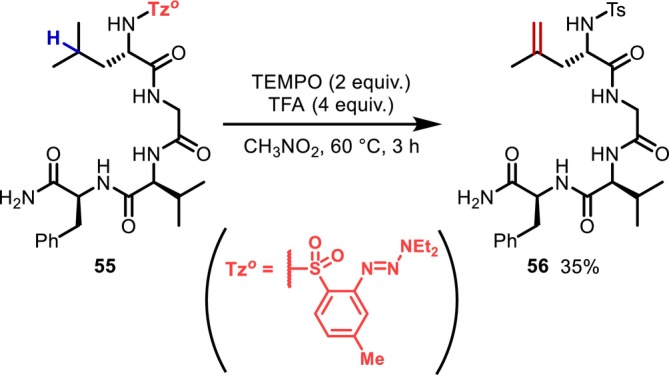
Triazene‐containing Ts group‐promoted desaturation of a Leu residue at the N‐terminal of a tetrapeptide through an intramolecular HAT process.

Furthermore, a few intramolecular HAT reactions other than 1,5‐HAT have been reported. In 2012, Baran demonstrated an intramolecular HAT reaction via the introduction of a triazene‐containing toluenesulfonyl (Tz^*o*^) group at the N‐terminal, achieving the desaturation of a Leu residue; this is the first report of an intramolecular HAT reaction that converts a peptide side chain (Figure 18) [55]. The Tz^*o*^ group is converted into a simple Ts group via the 1,7‐HAT reaction, together with the functionalization of the neighboring C(sp^3^)–H bond in the side chain.

**FIGURE 19:**
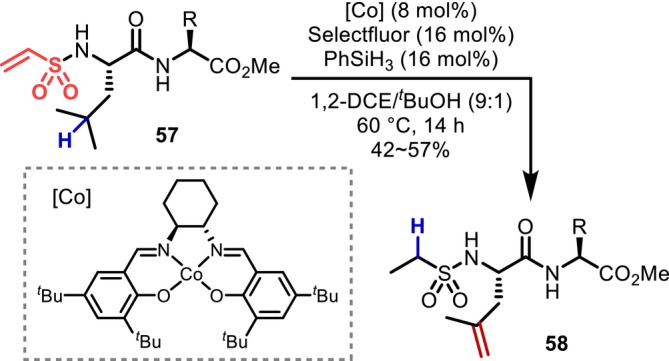
1,6‐HAT‐mediated desaturation of Leu residues at the N‐terminal of dipeptides catalyzed by a Co‐salen complex.

In 2022, the group of RajanBabu and Nagib achieved the desaturation of Leu residues on the N‐terminal through an unusual 1,6‐HAT process (Figure 19) [56]. A Co‐salen catalyst promotes MHAT to an alkene introduced as a vinyl sulfonamide at the N‐terminal of the peptides, resulting in an electron‐deficient alkyl radical that undergoes 1,6‐HAT. Subsequently, MHAT generates the terminal alkene and regenerates the Co‐hydride species. Although examples of reactions using C(sp^3^)‐C(sp^3^) HAT are rare, the authors suggest that the key to this reaction is the kinetic and thermodynamic destabilization of the alkyl radical by the adjacent sulfonyl group.

### 2.3 Directed, Metal‐Catalyzed C(sp^3^)–H Activation

**FIGURE 20:**
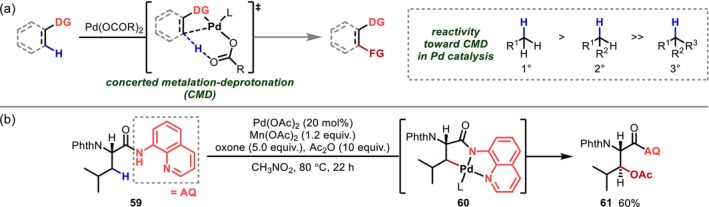
Pd‐catalyzed C(sp^3^)–H activation. (a) CMD mechanism. (b) β‐C(sp^3^)–H acetoxylation using an AQ directing group.

The chemistry of metal‐catalyzed C–H activation has remained a hot topic in synthetic organic chemistry since Murai et al.'s [57] report on aromatic C–H alkylation catalyzed by Ru complexes in 1993. Various transition metals have been employed as catalysts for C–H activation; among these, Pd‐based catalysts are the most widely used because they can activate even C(sp^3^)–H bonds via an inner‐sphere concerted metalation‐deprotonation (CMD) mechanism (Figure 20a) [58, 59, 60]. Although the assistance of directing groups is essential for efficient and site‐selective Pd‐catalyzed C–H activation, the reaction essentially only cleaves sterically less hindered primary and secondary C–H bonds, in contrast to the HAT chemistry described in Section 2.1, which preferentially targets tertiary C–H bonds, and can be considered a complementary approach.

The key to the successful activation of C–H bonds via this approach is the proximity of the target C–H bond to the Pd center. In general, reactions forming a five‐ or six‐membered palladacycle are preferred; the use of a bidentate directing group that can strongly capture Pd and fix the conformation of the Pd complex is extremely effective from this perspective. Aminoquinoline (AQ) and picolinamide (PA) are extremely important directing groups that are commonly used in various transformations involving the C–H activation and C–H arylation of AQ‐ or PA‐containing substrates; the report of Daugulis from 2005 [61, 62] represents a pioneering study in this field. AQ can be introduced into both amino groups and carboxamides, making it easier to apply to amino acid structures, and indeed, Corey has accomplished the acetoxylation and arylation of the β‐position of aliphatic side chains (alanine [Ala] and [Leu]) of amino acids by introducing AQ as a carboxamide [63] (Figure 20b). It should also be noted here that Val, which features a tertiary C–H bond at the β‐position, and *t*‐leucine (Tle), which does not contain a β‐C–H bond, undergo γ‐C–H activation via six‐membered palladacycles.

Subsequently, numerous reactions for the derivatization of amino acids through C–H activation were reported [64, 65]. However, they were not applied to the conversion of peptides until considerably later, probably because peptides contain multiple coordination sites including amide bonds, making a prediction of the coordination patterns of Pd complicated. In this context, Carretero reported in 2013 the C–H activation in peptides [66], followed by Yu in 2014 [67]. Since then, numerous reactions using native functional groups in peptides, such as main‐chain amides, terminal carboxyl or amino groups, as well as polar functional groups in side chains, as directing groups have been reported, sometimes with the introduction of AQ or PA at the peptide terminal to promote the reaction at the desired reaction site. These transformations are described in detail below, categorized by the side‐chain reaction site, which is closely related to the strategy.

#### 2.3.1 β‐C(sp^3^)–H Activation

β‐C–H Bonds in amino acid side chains are often located in positions where a five‐membered palladacycle can be formed through their activation, directed by main‐chain functional groups. Therefore, although the first report of C–H activation in peptides by Carretero occurs at the γ‐position [66], reactions at the β‐position are generally preferred in the absence of specific complicating factors such as a lack of C–H bonds that can react at the β‐position.

**FIGURE 21:**
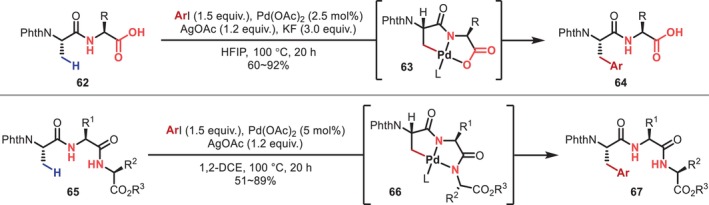
β‐C(sp^3^)–H Arylation reactions of peptide N‐terminal side chains promoted by the bidentate coordination of a native carboxylic acid and amide in the main chain.

In 2014, Yu reported the C–H arylation at the β‐position of the aliphatic side chain of the N‐terminal residue of a dipeptide (**62**) in which the C‐terminal carboxyl group is unprotected (Figure 21) [67]. In this reaction, C(sp^3^)–H activation is effectively promoted by the bidentate coordination of the native carboxyl group and the main‐chain amide. The authors also reported that even when the C‐terminal carboxyl group is protected, multiple main‐chain amides enable bidentate coordination to the Pd‐based catalyst in oligopeptides (**65**), resulting in similar C(sp^3^)–H arylation and acetoxylation in tripeptides and tetrapeptides. Phthaloyl (Phth) protecting groups were used in all reactions, presumably to inhibit the involvement of the N‐terminus in coordination to the Pd center. Subsequently, using the same strategy, Yu and Dong reported β‐C–H alkynylation reactions in 2017 [68] and β‐C–H alkenylation reactions in 2024 [69], respectively.

**FIGURE 22:**
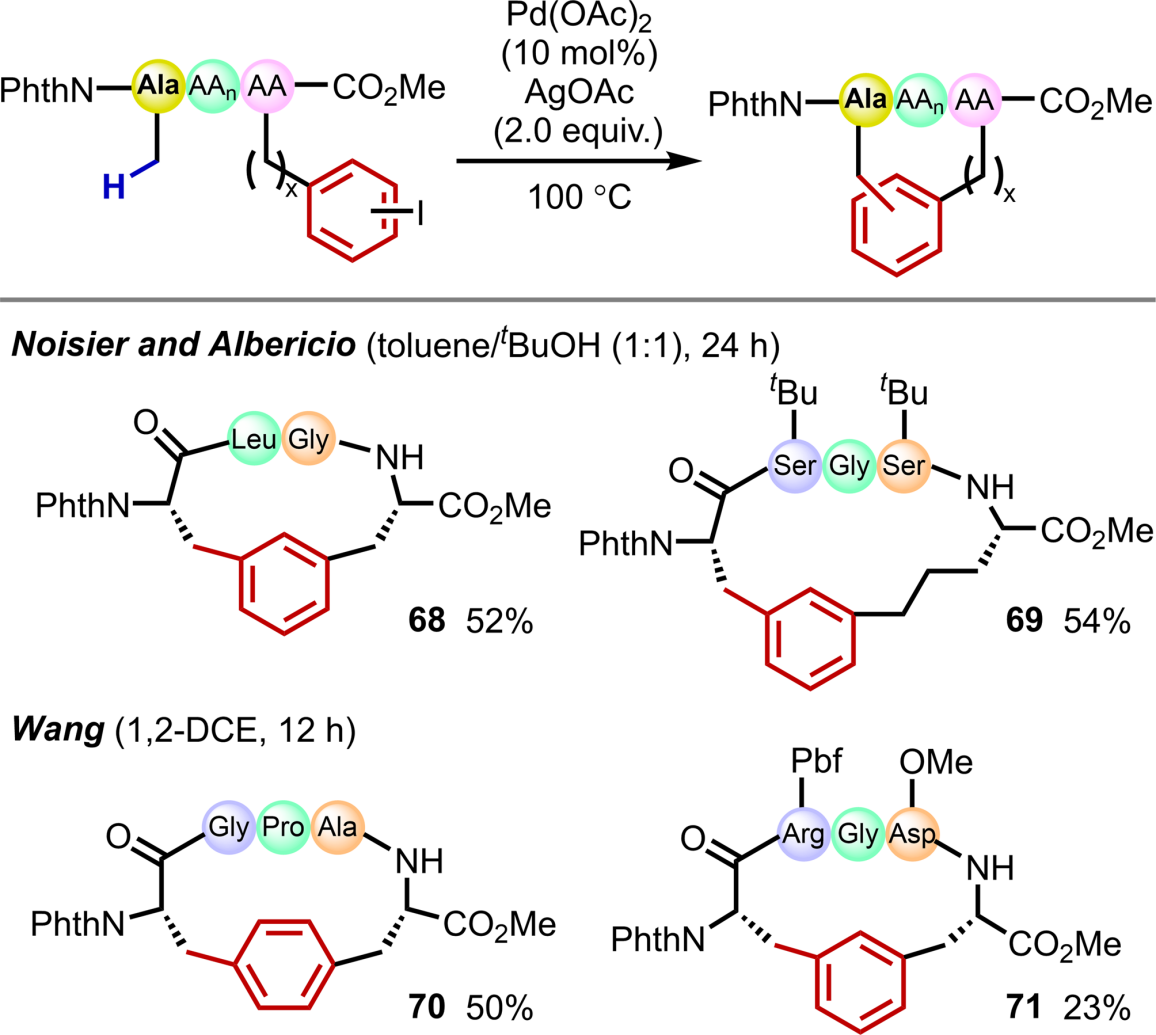
Synthesis of macrocyclic peptides via intramolecular β‐C(sp^3^)–H arylation at the N‐terminal side chains.

In 2017, the groups of Noisier and Albericio and that of Wang independently reported the synthesis of peptide macrocycles via intramolecular C(sp^3^)–H arylation (Figure 22) [70, 71]. Macrocyclic structures in peptides have become an essential element in mid‐sized peptide drug discovery [72, 73]. By introducing iodophenyl groups three or four residues away from the reactive N‐terminal residue, they successfully synthesized macrocyclic peptides through C–H activation directed by multiple amides in the main chain followed by cyclization. Whether the cyclization proceeds smoothly depends mostly on the nature and number of residues between the two residues to be cyclized. *Meta*‐substituted iodophenyl groups generally provide good results, probably due to conformational considerations during the reaction.

**FIGURE 23:**
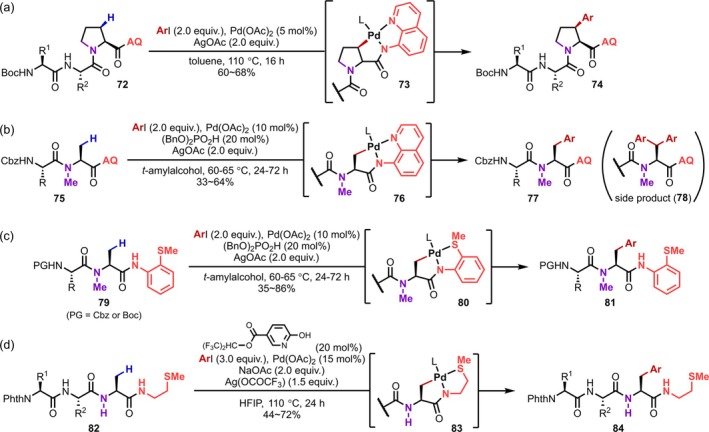
β‐C(sp^3^)–H Activation of peptide C‐terminal side chains promoted by the introduction of a bidentate directing group on the C‐terminus. (a) AQ‐directed β‐arylation of Pro residues. (b) AQ‐directed β‐arylation of *N*‐Me‐Ala residues. (c) Thioether‐directed β‐arylation of *N*‐Me‐Ala residues. (d) Thioether‐directed β‐arylation of simple Ala residues.

In contrast to the successful reactions at the N‐terminus promoted by native directing groups in peptides, it is difficult to achieve C(sp^3^)–H activation at the side chains of the C‐terminal residue in peptides using native functional groups, as the functional groups are not in the appropriate position for bidentate coordination. In 2016, Kazmaier introduced an AQ amide at the C‐terminus to achieve β‐C(sp^3^)–H arylation at a Pro residue on the C‐terminus (Figure 23a) [74]. This method allows using carbamoyl‐based protecting groups such as Boc and benzyloxycarbonyl (Cbz) groups, which are commonly used in peptide synthesis, on the N‐terminus of the peptides. In 2018, the reaction was applied to the β‐C(sp^3^)–H arylation of the *N*‐methyl amino acid side chain on the C‐terminus (Figure 23b) [75]. In 2020, Chen reported the synthesis of macrocyclic peptides by applying this method to an intramolecular reaction in which 9‐ to 27‐membered rings are formed [76]. In intermolecular arylation reactions using an AQ amide as a directing group, a significant amount of diarylated products was observed due to overreaction. In 2019, Kinsinger and Kazmaier [77] obtained the monoarylation products by using less reactive 2‐methylthioanilide as the directing group (Figure 23c). Additionally, the amide closest to the C‐terminus must be a tertiary amide such as *N*‐methylamide in order to avoid undesired coordination of the amide to the Pd center [74, 75, 76, 77]. To overcome this problem, Yao developed in 2021 the use of thiomethylethyl amide as a new bidentate directing group and succeeded in circumventing the aforementioned restrictions, albeit that this reaction has only been applied to peptides with phthaloyl protection on the N‐terminus (Figure 23d) [78].

Up to this point, we have discussed the β‐C(sp^3^)–H activation of side chains at the N‐ or C‐termini with the assistance of main‐chain functional groups as directing groups for bidentate coordination. As an alternative approach, C(sp^3^)–H activation, in which a native functional group in the side chain of the residue adjacent to the reacting aliphatic side chain acts as one of the directing groups for bidentate coordination, has also been intensively investigated.

**FIGURE 24:**
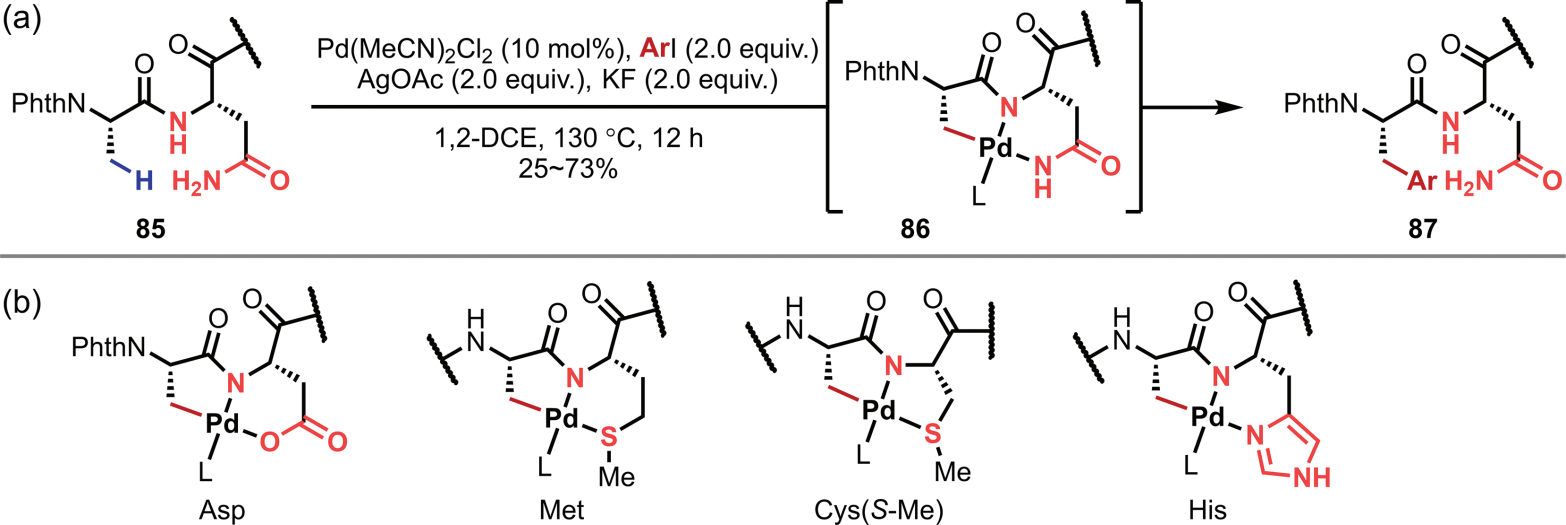
Site‐selective β‐C(sp^3^)–H activation of the Ala residue directed by side‐chain functional groups. (a) Direction of primary amide in Asn residues. (b) Coordination patterns with side‐chain functional groups.

In 2020, Ackermann reported β‐C(sp^3^)–H arylation reactions using a primary amide of the asparagine (Asn) residue as a directing group, in which the Ala residue adjacent to the N‐terminal side of the Asn residue reacts selectively (Figure 24a) [79]. In 2024, Dong also achieved alkynylations based on the same approach [80]. Subsequently, this strategy was applied to carboxy groups in aspartic acid (Asp) residues (2021) [81], sulfide groups in methionine (Met) residues (2022) [82, 83], sulfide groups in *S*‐methylated Cys residues (2024) [84], and imidazole groups in histidine (His) residues (2024) [85] (Figure 24b). In all cases, it was assumed that the functional groups of the side‐chain and the main‐chain amide at the N‐terminal side of the residue coordinate to Pd in a bidentate fashion. Even in cyclic peptides or long oligopeptides, the Ala residue adjacent to the N‐terminal side of the residue with the directing functional group reacts selectively; however, the reaction appears to be limited to Ala residues, and therefore, the scenarios in which it is applicable are rather limited. In 2022, Chen also applied the Met‐directed C(sp^3^)–H arylation to the synthesis of macrocyclic peptides [83].

#### 2.3.2 γ‐C(sp^3^)–H Activation

**FIGURE 25:**
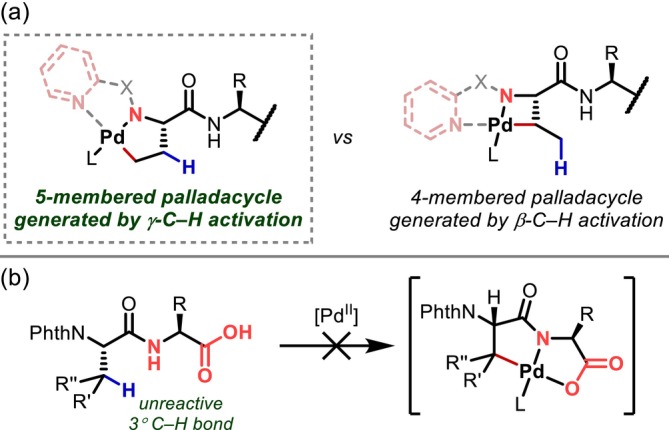
Requirements for γ‐C(sp^3^)–H activation. (a) 5‐Membered palladacycle vs 4‐membered one. (b) Lack of reactive β‐C(sp^3^)–H bonds.

As mentioned above, the β‐position is generally the position most susceptible to C–H activation in the peptide side chain. The situations where γ‐C(sp^3^)–H activation proceeds are limited to the following: (1) when the amino group at the N‐terminus acts as a directing group and the C–H bond on the N‐terminal residue is activated (Figure 25a), or (2) when there are no primary or secondary C–H bonds that can react at the β‐position, that is, in the case of Val, isoleucine (Ile), threonine (Thr), and Tle residues (Figure 25b).

**FIGURE 26:**
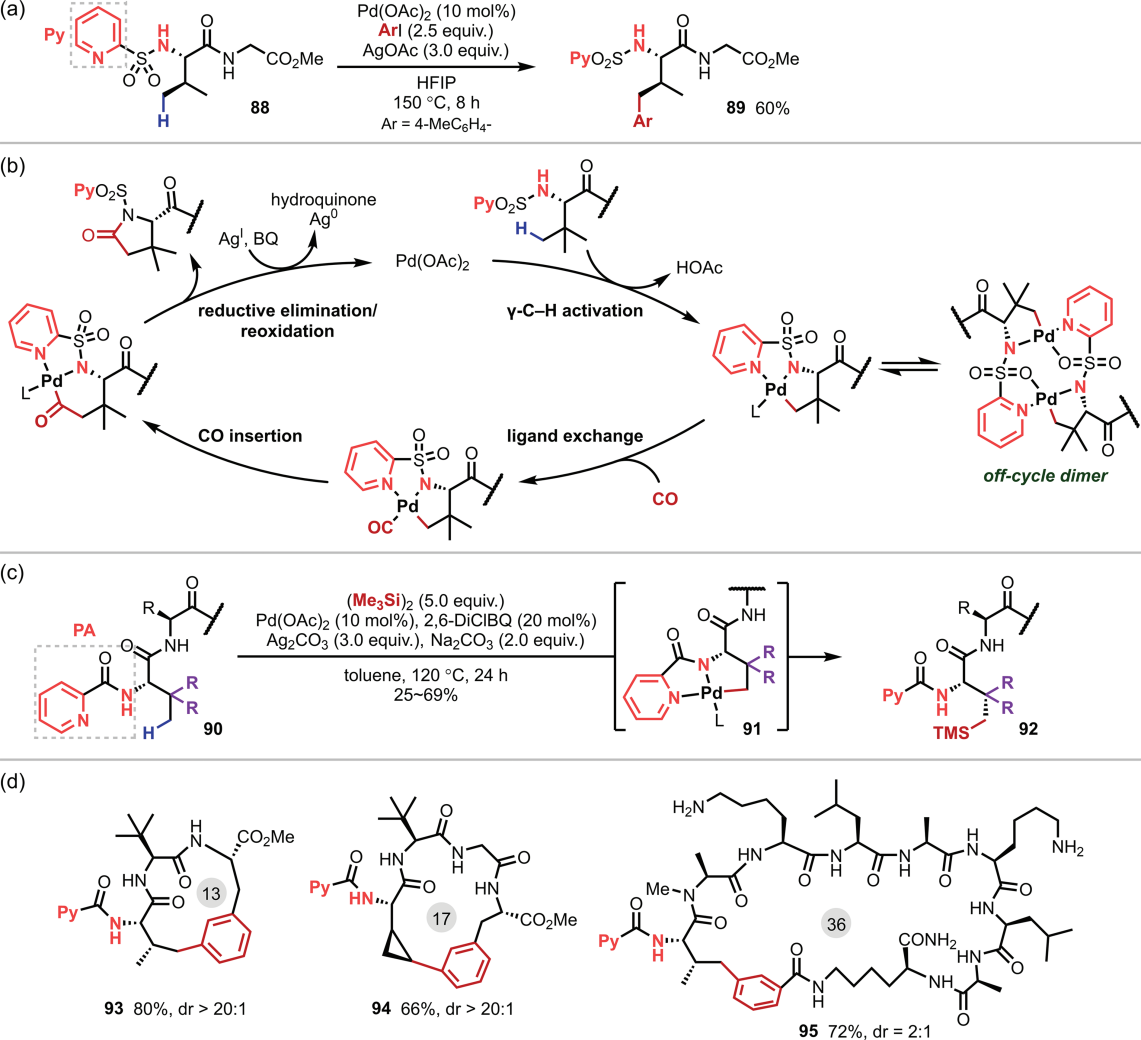
Activation of γ‐C(sp^3^)–H bonds on the N‐terminal residue promoted by 2‐pyridylsulfonamide and PA directing groups on the N‐terminus. (a) 2‐Pyridylsulfonamide‐directed γ‐arylation. (b) Catalytic cycle for γ‐carbonylative cyclizations. (c) PA‐directed γ‐silylations. (d) Substrate scope for macrocyclizations.

In 2013, Carretero reported the arylation of the γ‐C(sp^3^)–H bonds of Val and Ile residues at the N‐terminus by introducing 2‐pyridylsulfonyl as a directing group on the N‐terminal amino group [66]; β‐arylation, which would presumably proceed via a four‐membered palladacycle, did not occur, and only the γ‐arylation product produced via a five‐membered palladacycle was obtained, even when an 2‐aminobutyric acid (Abu) residue, which contains C–H bonds that are reactive in the Pd‐mediated reaction at both the β and γ positions, was used (Figure 26a). In 2016, the same group applied this approach to a carbonylative cyclization reaction using Mo(CO)_6_ to afford a lactam [86]. Initially, it was thought that a bimetallic complex consisting of two peptide substrates and two Pd centers that was isolated and characterized by X‐ray crystallography plays an important role in this transformation [66]. However, a detailed subsequent mechanistic analysis revealed that this bimetallic complex is an off‐cycle species and that the reaction proceeds instead via mononuclear Pd complexes (Figure 26b) [86]. In 2019, Shi reported the γ‐silylation of the N‐terminal residue via the introduction of a PA motif to the N‐terminus (Figure 26c) [87]. Both 2‐pyridylsulfonamide and PA can be removed using reductants such as Zn under acidic conditions. Subsequently, Chen reported the synthesis of macrocyclic peptides via γ‐C–H arylation using N‐terminal PA as a directing group [88]. The authors obtained 29 macrocyclic peptides with 13‐ to 36‐membered rings in good yield (Figure 26d).

**FIGURE 27:**
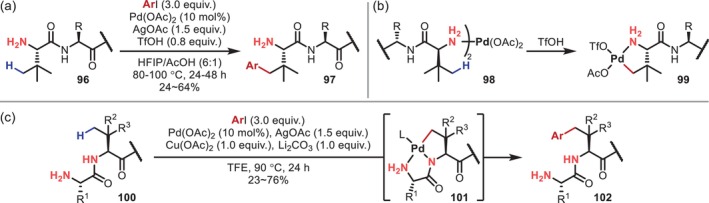
Activation of γ‐C(sp^3^)–H bonds promoted by an unprotected N‐terminal amino group. (a) γ‐Arylation on N‐terminal residues. (b) Bidentate DG‐promoted γ‐arylation adjacent to N‐terminal residues.

In 2019, Yao reported the γ‐C(sp^3^)–H arylation of an N‐terminal Tle residue in a peptide (**96**) with an unprotected N‐terminal amino group as a directing group (Figure 27a) [89]. The use of the N‐terminal amino group as a directing group may lead to the formation of inactive off‐cycle complexes due to bidentate coordination with neighboring carbonyl groups. In this context, the authors suggested that the key to this reaction may be the introduction of a bulky structure such as Tle. Moreover, regarding the effect of trifluoromethanesulfonic acid (TfOH), it was later proposed that the bis(amine)‐Pd complex (**98**) involving two peptide amines decomposes to afford more active mono(amine)‐Pd complexes (**99**), thus promoting γ‐C–H activation (Figure 27b) [90]. The same authors also reported that in the absence of an acid, the activation of the γ‐C–H bond of the residue adjacent to the N‐terminus proceeds due to bidentate coordination with the adjacent amide (Figure 27c) [90].

**FIGURE 28:**
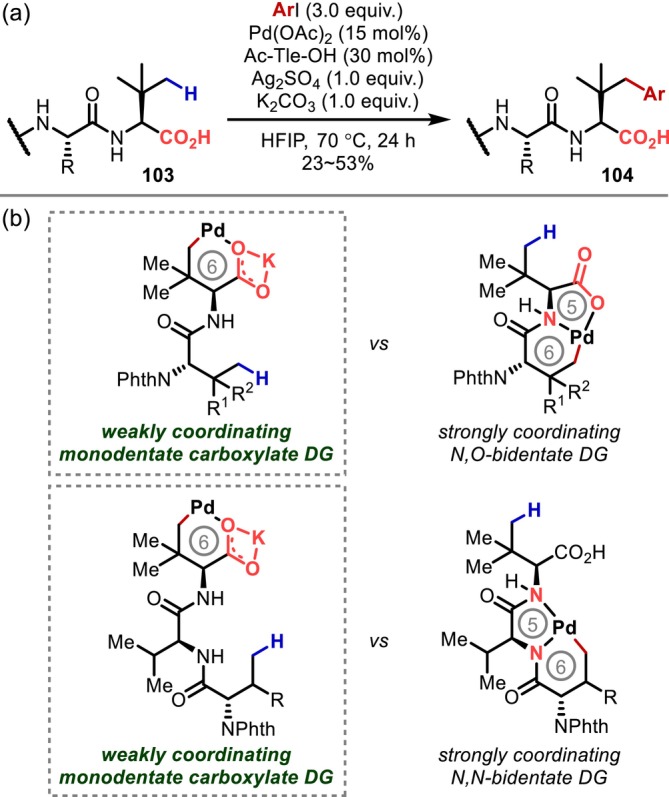
(a) Activation of γ‐C(sp^3^)–H bonds on a Tle residue on the C‐terminal residue directed by an unprotected carboxylic acid on the C‐terminus. (b) Competitive coordination patterns.

In 2020, Shi reported the γ‐C(sp^3^)–H arylation of C‐terminal Tle using an unprotected carboxylic acid on the C‐terminus as a directing group (Figure 28a) [91]. The proposed coordination pattern for this approach is the same as that reported by Yu [67]; however, in Shi's study, the residue adjacent to the C‐terminus, which is the reaction site in Yu's report, was modified to eliminate the C–H bond that could react at the β‐position. Consequently, the only possibility for bidentate coordination is a reaction with the γ‐C–H bonds on the residue to form a 5,6‐fused metallacycle. The authors proposed that this is less favorable than a monocyclic six‐membered metallacycle with the γ‐position of the C‐terminal residue, resulting in γ‐C–H arylation on the C‐terminus, albeit that this reaction was only used for peptides containing Tle on the C‐terminus (Figure 28b).

#### 2.3.3 δ‐C(sp^3^)–H Activation

**FIGURE 29:**
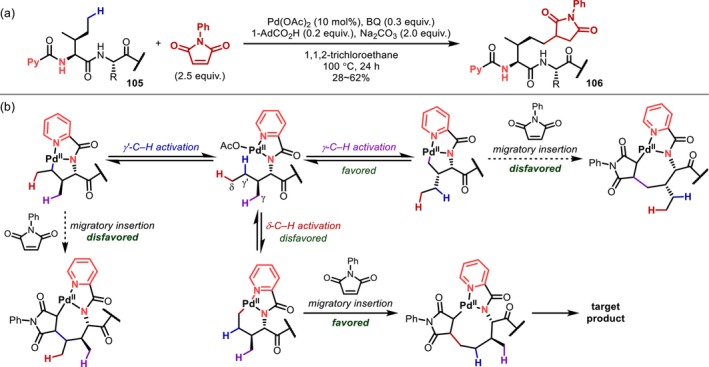
(a) δ‐C(sp^3^)–H Alkylation using maleimide. (b) Explanation for δ‐selectivity.

The δ‐C(sp^3^)–H bonds of side chains are located far from the main chains that serve as the directing group. Therefore, in the C–H activation of Ile with the N‐terminal amino group as the directing group, γ‐C–H activation to provide a five‐membered palladacycle is preferred over δ‐C–H activation to form a six‐membered palladacycle, which is kinetically less favorable [87]. However, in 2018, Shi reported that C(sp^3^)–H alkylation incorporating maleimide preferentially proceeds at the δ‐position rather than the γ‐position of the Ile residue in peptides (**105**) containing a PA directing group on the N‐terminus (Figure 29a) [92]. In mechanistic studies, the authors demonstrated that both the γ‐ and δ‐C–H activation are reversible using deuterium‐labeling experiments and proposed that the selective formation of δ‐alkylated compounds is due to the migratory insertion of maleimide proceeding exclusively from the six‐membered metallacycle formed by δ‐C–H activation (Figure 29b). Nevertheless, this reaction is quite exceptional, and there is no doubt that C–H activation at the δ‐position of the peptide side chain is an extremely limited and difficult transformation.

### 2.4 Transformation of C(sp^2^)–H Bonds in Aromatic Residues

Although the description of aromatic amino acid residues in peptides as “chemically inert” may be somewhat controversial, the conversion of their C(sp^2^)–H bonds is often much more difficult than FGT of heteroatom‐based functional groups. Therefore, this section provides a brief overview of the transformations of C(sp^2^)–H bonds in aromatic amino acids organized according to residue (tryptophan [Trp], Tyr, Phe, and His).

#### 2.4.1 Conversion of C(sp^2^)–H Bonds in Trp Residues

**FIGURE 30:**
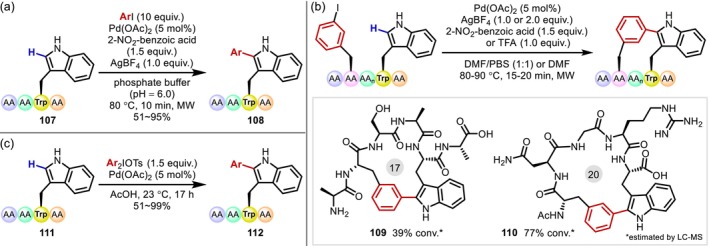
Pd‐catalyzed C2‐arylation of the indole ring of Trp residues in peptides. (a) Intermolecular reaction. (b) Intramolecular cyclization to provide macrocyclic peptides. (c) Low‐temperature arylation with hypervalent iodine (III) reagent.

The indole ring in the Trp residue is the aromatic ring most frequently targeted in the chemical modification of peptides. Transition metal‐catalyzed C(sp^2^)–H activation on the Trp residues of peptides has been extensively explored since the first reports of Albericio and Lavilla on using Pd catalysis in 2010 [93]. In 2006, Sanford first reported a Pd‐catalyzed C(sp^2^)–H arylation at the 2‐position of indoles, in which a hypervalent iodine (III) reagent was used to facilitate a Pd(II)/Pd(IV) cycle [94]. The subsequent work of Lebrasseur and Larrosa [95] described *o*‐nitrobenzoic acid‐promoted C2‐arylation using iodoarenes as electrophiles. Inspired by these precedents, the C2‐arylation of indole rings in Trp and peptides containing Trp (**107**) was achieved for the first time in 2010 (Figure 30a) [93]. Although simple indole reacts smoothly even at room temperature under the catalytic conditions developed by Lebrasseur and Larrosa [95], the reaction of Trp residues requires high temperatures (150°C). In contrast, the reaction proceeds at lower temperature (80°C) in peptides, which the authors attributed to a stabilization of the intermediate complex by the peptide scaffold. In 2012, James applied this method to the macrocyclization of peptide analogues containing unusual motifs [96], before Albericio and Lavilla described a similar cyclization to staple “true” peptides [93]. The reaction exhibits remarkable functional‐group tolerance in the presence of unprotected serine (Ser), Asp, Asn, and arginine (Arg) residues, which could potentially poison transition metal catalysts (Figure 30b) [97]. Several examples of similar C2‐arylations have been reported since, including that of Ackermann, who used hypervalent iodine (III) at room temperature in aqueous media (Figure 30c) [98].

**FIGURE 31:**
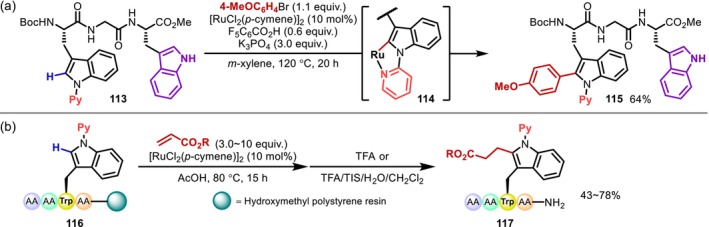
(a) 2‐Pyridyl group‐directed C2‐arylation of the indole ring of Trp residues in peptides. (b) C2‐Alkylation with electron‐deficient alkenes.

In 2017, Ackermann reported the C2‐arylation of Trp residues with a 2‐pyridyl directing group introduced at the 1‐position of the indole ring (Figure 31a) [99]. The directed reaction was also found to proceed preferentially even when another indole group without a directing group was present. Subsequently, the same group revealed that this approach is also applicable to resin‐supported peptides, albeit that the removal of the 2‐pyridyl group requires the strong electrophile methyl trifluoromethanesulfonate (MeOTf) and the generality of the method is still somewhat unclear (Figure 31b) [100].

**FIGURE 32:**
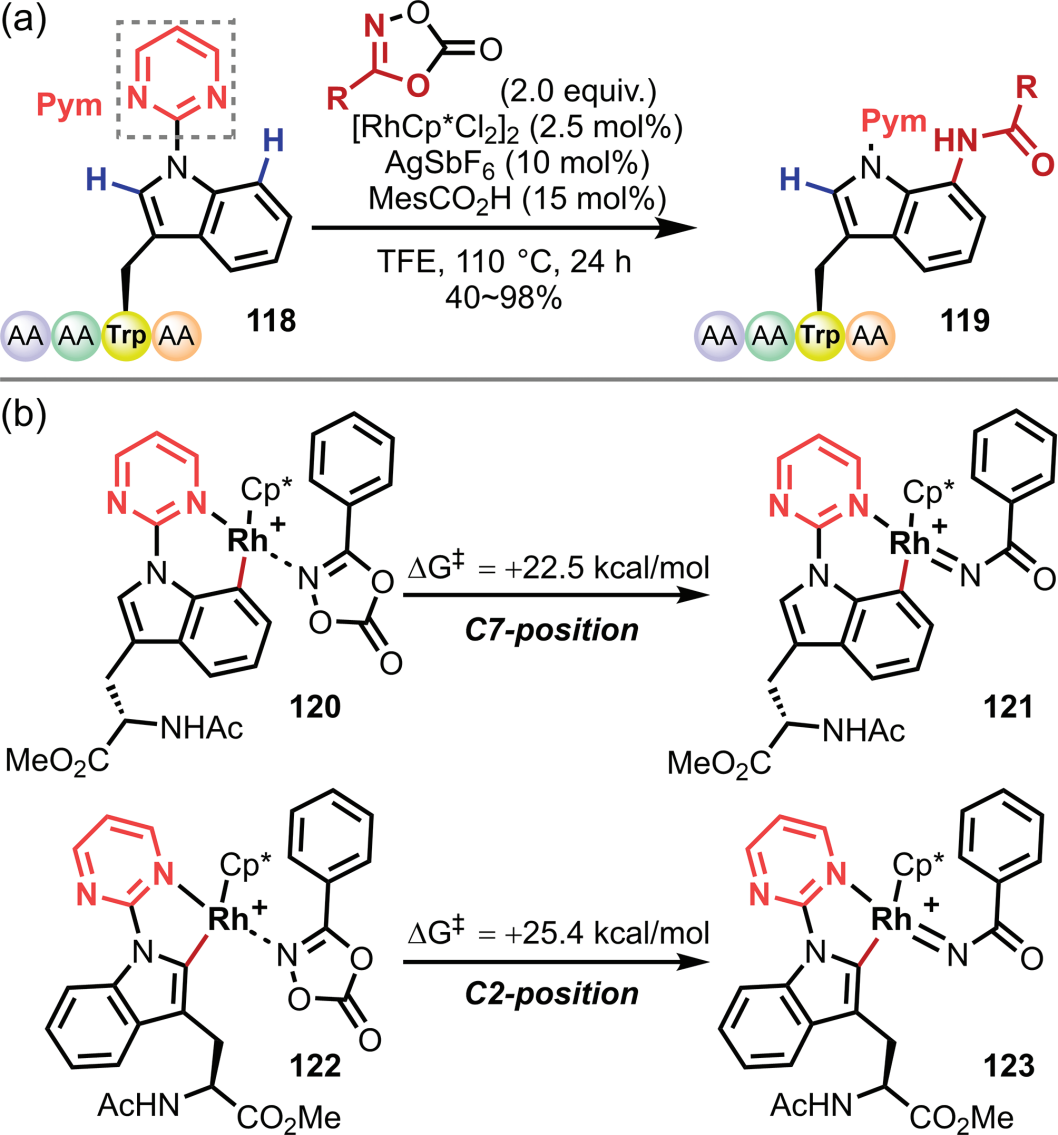
(a) Rh‐catalyzed C7‐amidation of 2‐pyrimidyl group‐containing Trp residues in peptides. (b) Explanation for C7‐selectivity.

A more noteworthy report of a reaction involving directing groups is the C(sp^2^)–H functionalization at the 7‐position of indoles. In general, indoles are highly reactive at the 2‐ and 3‐positions; in contrast, the conversion of C(sp^2^)–H bonds on the six‐membered ring (i.e., at the 4‐, 5‐, 6‐, and 7‐positions) is challenging due to both selectivity and reactivity issues. Against this background, Ackermann reported in 2020 that Rh‐catalyzed C(sp^2^)–H amidation proceeds preferentially at the 7‐position in a Trp residue with a 2‐pyrimidyl group and demonstrated the first C7‐functionalization of Trp in peptides (**118**) (Figure 32a) [101]. A deuterium‐labeling experiment showed that deuteration occurs at both the C2 and C7 positions, suggesting that the C7‐selectivity of this reaction is derived not from the C(sp^2^)–H activation step, but instead from the subsequent step (Figure 32b). This seems reasonable considering that the reaction at the C2 position proceeds selectively in the Ru‐catalyzed arylation using almost the same substrate [99].

**FIGURE 33:**
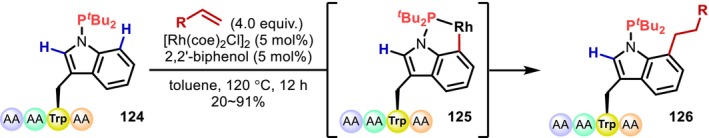
P(III)‐directed C7‐alkylation of Trp residues in peptides.

Subsequently, in 2022, Shi and Wang also reported a C7‐selective alkylation of an indole bearing a P(III) substituent at the 1‐position as the directing group (Figure 33) [102]. In this system, the reaction is not likely to proceed at the 2‐position, as this would require the formation of a four‐membered metallacycle. This is probably the reason for the excellent C7 selectivity. In addition, the P(III) directing group is rapidly removed under mild conditions via treatment with *N*,*N*‐diethylaminosulfur trifluoride (DAST) at low temperatures.

**FIGURE 34:**
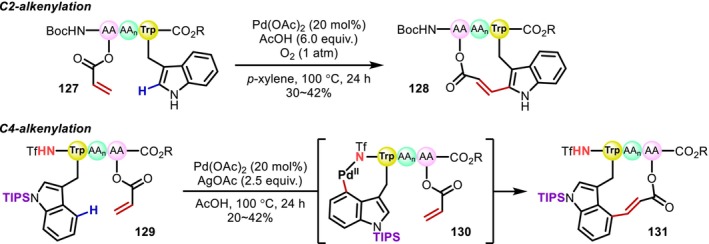
Macrocyclization of Trp‐containing peptides through Pd‐catalyzed C2‐ or C4‐alkenylation of indole rings with electron‐deficient alkenes.

In 2020, Wang reported a Pd‐catalyzed macrocyclization reaction of Trp‐containing peptides (**127** and **129**) via migratory insertion of electron‐deficient alkenes. In this system, the C(sp^2^)–H activation typically proceeds at the 2‐position, whereas for triisopropylsilyl (TIPS)–protected Trp residues at the N‐terminus with a trifluoromethanesulfonyl (Tf) protecting group, it proceeds selectively at the 4‐position (Figure 34) [103]. The C4‐alkylation of TIPS‐protected Trp monomers had already been reported by Jia in 2013 [104].

In terms of alternative approaches for the transformation of C(sp^2^)–H bonds in Trp residues, various C2 functionalizations involving the addition of electrophilic radicals to the 2‐position of indole followed by rearomatization taking advantage of the high nucleophilicity of the indole ring have been developed since the report of Stephenson in 2010 [105]. Among these, there are numerous reactions that exhibit excellent chemoselectivity based on the “high reactivity” of indole [106]. However, these are beyond the scope of this review, which aims at presenting methods for converting chemically inert peptides, and have thus been omitted here.

#### 2.4.2 Conversion of C(sp^2^)–H Bonds in Tyr Residues

**FIGURE 35:**
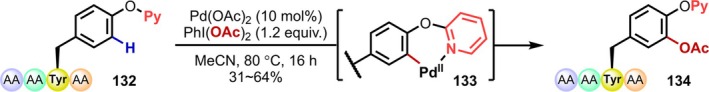
*O*‐2‐Pyridyl group‐directed Pd‐catalyzed C(sp^2^)–H activation of Tyr residues in peptides.

The electron‐rich phenol motif in the Tyr residue is “highly reactive” toward electrophilic substitution reactions, allowing smooth *ortho*‐halogenations followed by FGT to easily provide derivatives. In this context, Correa reported in 2023 the Pd‐catalyzed C(sp^2^)–H activation of Tyr residues in peptides (**132**) by introducing an *O*‐2‐pyridyl substituent as a directing group (Figure 35) [107].

In another approach, Tyr, which features an electron‐rich aromatic ring like Trp, has been functionalized in radical reactions with electrophilic species, which are also beyond the scope of this review.

#### 2.4.3 Conversion of C(sp^2^)–H Bonds in Phe Residues

**FIGURE 36:**
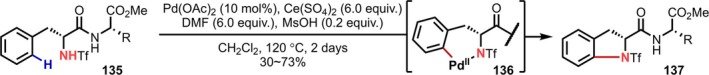
Pd‐catalyzed cyclization through C(sp^2^)–H activation of a Phe residue directed by the Tf‐protected amino group of the N‐terminus.

Unlike Trp and Tyr residues, the Phe residue does not have a heteroatom that can act as a reactive site or promote reactivity, making its selective conversion more difficult relative to that of the aliphatic amino acids described above. Therefore, other than approaches that dearomatize the benzene ring, transition metal‐catalyzed C(sp^2^)–H activation is the only option to convert the side chains of the Phe residue. Despite the remarkable development of transition metal‐catalyzed C–H activation chemistry, it has not been applied to the diversification of Phe residues in peptides until the report of Shi [108] on the Pd‐catalyzed cyclization involving the Phe residue on a Tf‐protected N‐terminus to afford proline analogues in 2015 (Figure 36).

**FIGURE 37:**
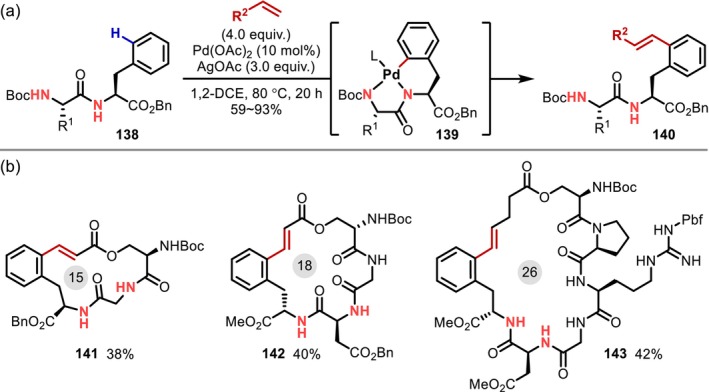
(a) Pd‐catalyzed C(sp^2^)–H alkenylation of the Phe residue directed by native amides of the peptide main chain. (b) Substrate scope for macrocyclizations.

In 2018, Xuan reported a similar cyclization of dipeptides promoted by the bidentate coordination of an amide in the peptide main chain and a carbamate or amide group on the N‐terminus [109]. In the same year, Wang reported the Pd‐catalyzed C(sp^2^)–H alkenylation of the Phe residue with the assistance of the native amides of the peptide main chain as directing groups (Figure 37a) [110]. In this reaction, C–H activation at the 2‐position of the benzene ring of the Phe residue proceeds via bidentate coordination with two amides on the N‐terminal side from the Phe residue, yielding a 5,6‐bicyclic palladacycle (**139**). The authors demonstrated macrocyclization involving a Phe residue and a preinstalled alkene to afford 15‐ to 26‐membered rings (Figure 37b). Around the same time, Cross reported the C–H alkenylation of Phe residues in peptides using a similar strategy [111], whereas Zheng and Song also described alkynylation in addition to alkenylation [112].

**FIGURE 38:**
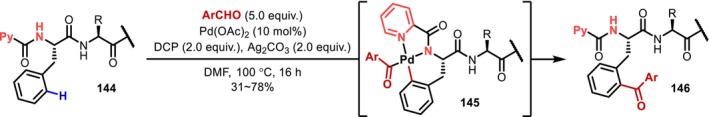
PA‐directed Pd‐catalyzed C(sp^2^)–H acylation of the Phe residue on the N‐terminus.

In 2019, Segundo and Correa [113] demonstrated a Pd‐catalyzed C(sp^2^)–H acylation of the Phe residue on the N‐terminus by introducing a PA directing group (Figure 38). Later, this approach was applied to chalcogenation [114] and coupling with benzoquinone [115, 116].

**FIGURE 39:**
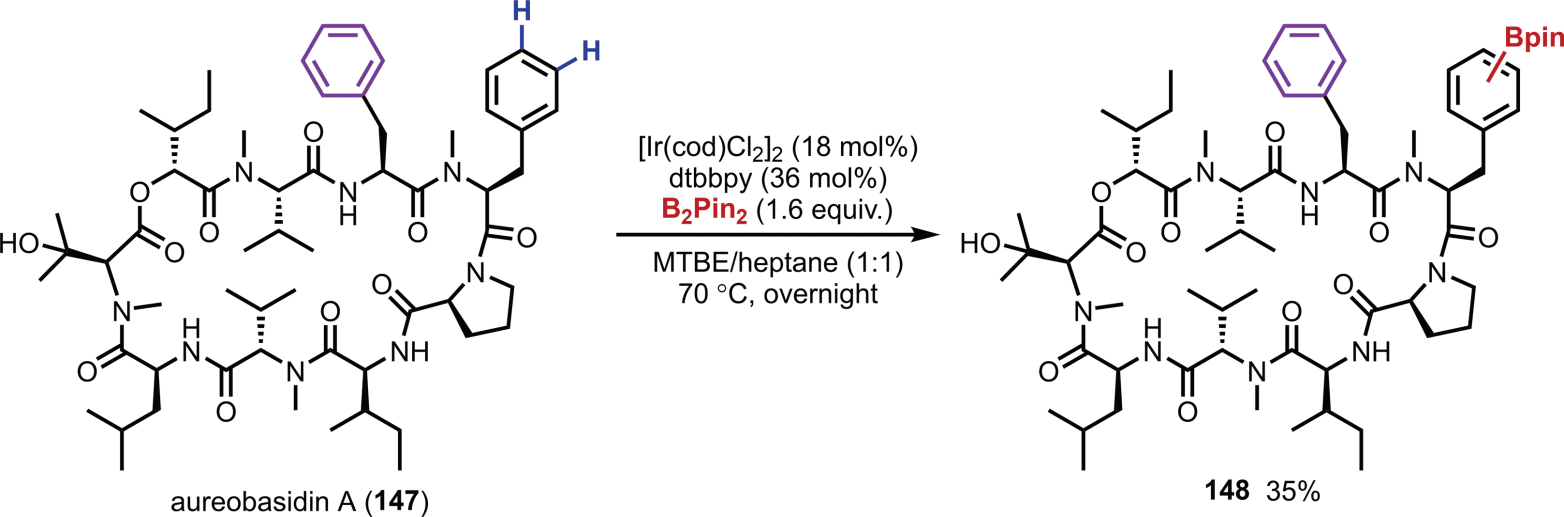
Ir‐catalyzed C(sp^2^)–H borylation of the Phe residue of AbA.

Another noteworthy report was published in 2015 by Elhammer, who described the Ir‐catalyzed C(sp^2^)–H borylation of a Phe residue in aureobasidin A (AbA, **147**), a macrocyclic depsipeptide natural product that exhibits antifungal activity (Figure 39) [117]. Interestingly, the Ir‐catalyzed C(sp^2^)–H borylation proceeded selectively at only one of the two Phe residues, and subsequent Suzuki coupling reactions yielded various substituted compounds, whereas the selectivity between *meta* and *para* substitution on the benzene ring was poor.

**FIGURE 40:**
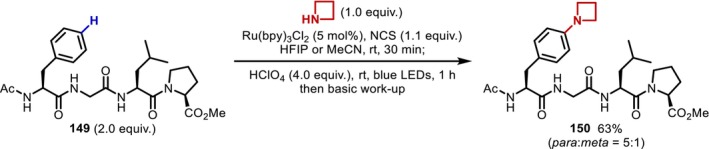
Photocatalytic C(sp^2^)–H amination of Phe residues in peptides.

Finally, we close this section by introducing a recent report by Leonori [118] on the use of ammonium radicals to functionalize Phe residues. C(sp^2^)–H amination promoted by electrophilic radicals is generally considered to be a challenging transformation due to the difficulties associated with suppressing over reaction, as the amination products are more electron‐rich than the starting material. Against this background, Leonori discovered that the generation and reaction of a nitrogen radical under acidic conditions successfully proceed at the *para* position of a benzene ring and applied the transformation to a Phe residue‐containing tetrapeptide (**149**) (Figure 40).

#### 2.4.4 Conversion of C(sp^2^)–H Bonds in His Residues

**FIGURE 41:**
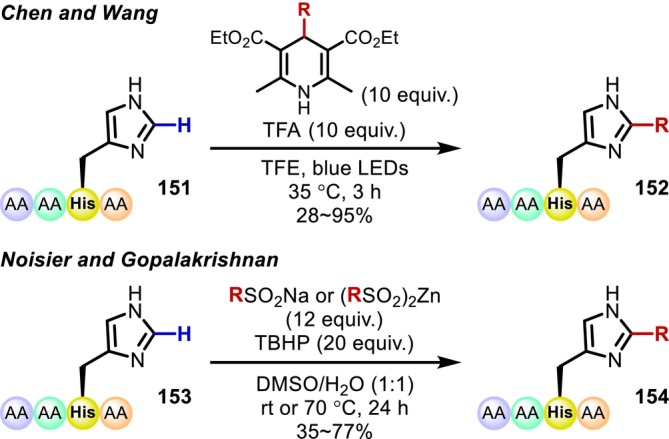
His‐selective C(sp^2^)–H alkylation.

Transformations of the C(sp^2^)–H bonds of the imidazole ring in His residues are extremely rare. The addition reactions of alkyl radicals to the imidazole ring that have been reported simultaneously by the group of Chen and Wang [119] and that of Noisier and Gopalakrishnan [120] are notable examples of such reactions. As alkyl radicals are nucleophilic, they selectively react with the electron‐deficient imidazole in the His residue even in the presence of electron‐rich aromatic amino acid residues such as Trp and Tyr. The authors demonstrated that the reaction proceeds even in peptides with dozens of residues (Figure 41).

## 3 Modification of Amides in the Peptide Main Chain

The main chain of a peptide serves as the foundation for its three‐dimensional structure, and thus, structural modifications of the amide groups inevitably have a significant impact on the properties and functions of the peptide. Therefore, replacement of the main‐chain amides with other motifs to improve the function of peptides has frequently been attempted [121]. However, synthetic chemistry approaches have traditionally relied on extending peptide chains beginning from the unit containing the desired modification sites, simply because reaction with the peptide main‐chain amides, which are neither sufficiently electrophilic nor nucleophilic, is difficult. To overcome this challenge, tremendous efforts have recently been devoted to the development of reactions that can be used for the late‐stage modification of peptides. The following sections provide an overview of these reactions, categorized by reaction type.

### 3.1 *N*‐Carbon Substitution

The introduction of carbon substituents on the amide nitrogen of the peptide main chain drastically alters the three‐dimensional structure of the peptides and is frequently used to fix the peptide in specific conformations [122]. Furthermore, the low membrane permeability of peptides due to their high hydrophilicity is problematic in medicinal chemistry; therefore, the introduction of *N*‐alkyl substituents on the peptide main chain to enhance lipophilicity and metabolic stability has recently become an essential element in midsized peptide drug discovery [123].

The synthesis of *N*‐alkylated peptides, similar to that of conventional peptides, is generally performed using Fmoc solid‐phase peptide synthesis (SPPS). However, the presence of bulky *N*‐alkyl amino acids often significantly decreases the reaction efficiency of dehydrative condensations. Alternatively, the late‐stage *N*‐alkylation of constructed peptides, which is also effective for diversifying peptide compound libraries, has also been explored.

**FIGURE 42:**
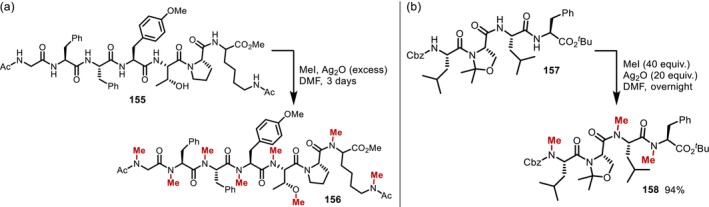
*N*‐Alkylation of peptides using Ag_2_O/MeI. (a) Permethylation of a heptapeptide. (b) Permethylation of a tetrapeptide monomer for the synthesis of IB‐01212.

The earliest example of the *N*‐methylation of peptides was reported by Das et al. in 1967 [124]; there, permethylation of a heptapeptide (**155**) was achieved using silver oxide and methyl iodide. However, this method requires a very large excess of silver oxide and methyl iodide (as a cosolvent), and the purity of the product, including the possibility of epimerization, was not mentioned (Figure 42a). Subsequently, the *N*‐methylation protocol was used to determine the position of the *N*‐methyl group in *N*‐methylated peptides via mass spectrometry [125, 126]. Later, Oishi and Fujii synthesized the antiproliferative dimeric depsipeptide IB‐01212 by applying the Ag‐mediated *N*‐methylation protocol to a tetrapeptide monomer (**157**) (Figure 42b) [127].

**FIGURE 43:**
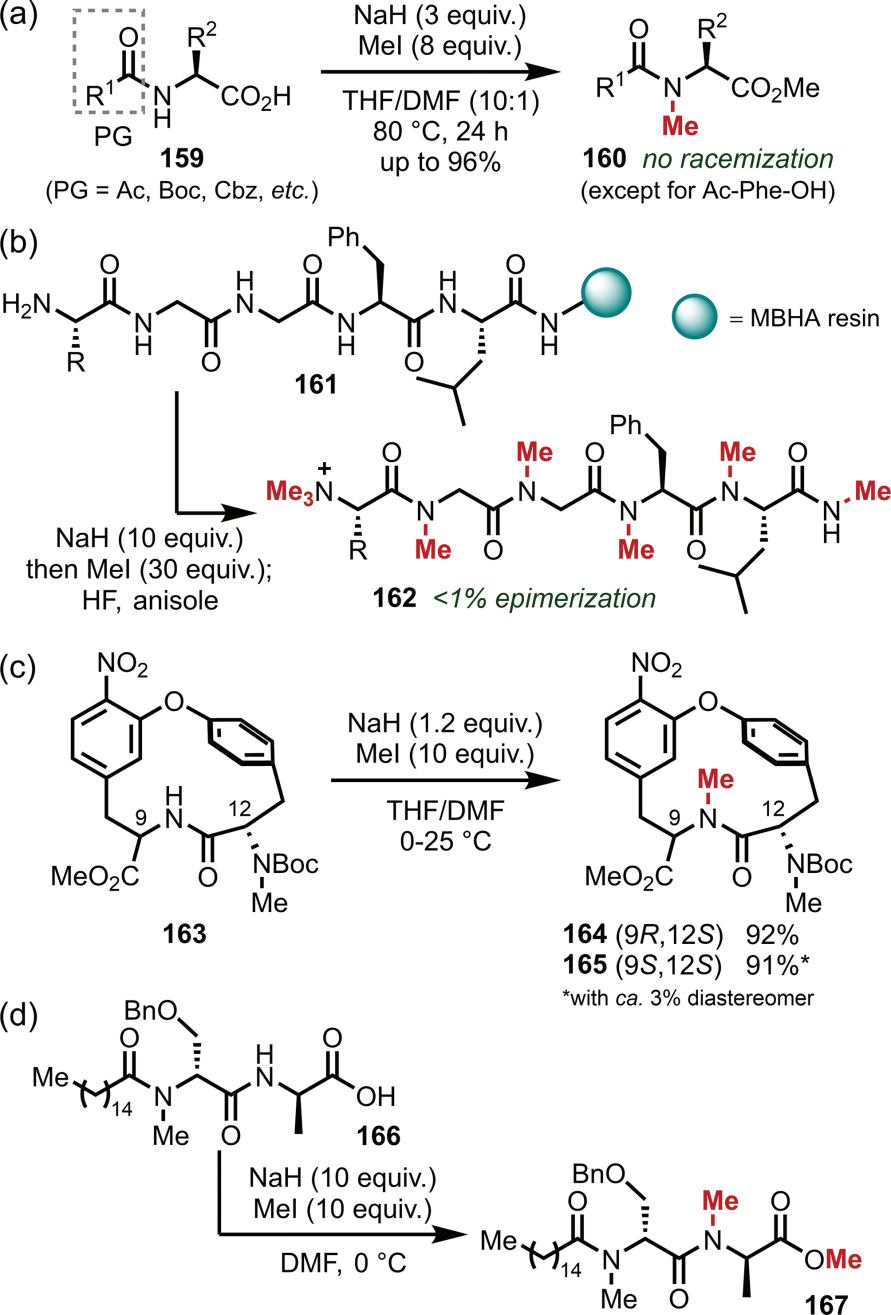
NaH‐mediated *N*‐alkylation of peptides. (a) *N*‐Methylation of *N*‐protected amino acids. (b) Permethylation of peptides on resins. (c) *N*‐Methylation for the synthesis of *N*‐methylcycloisodityrosine. (d) *N*‐Methylation of cyclic peptide units in the synthesis of antibiotic arylomycins.

As an alternative to this economically inefficient protocol requiring large amounts of Ag salts, *N*‐methylation using strong bases has also been investigated. In 1971, Coggins and Benoiton [128] reported that *N*‐methylation conditions using sodium hydride and methyl iodide at 80°C, which had previously been used for simple amides by Fones [129], provided access to *N*‐methylated amino acids (**160**) (Figure 43a). Despite the strong concern about α‐racemization under strongly basic conditions, the authors mentioned that, surprisingly, racemization was not observed except in the case of *N*‐Ac‐Phe‐OH. Subsequently, in 1994, Houghten achieved the permethylation of peptide chains (**161**) supported on a solid phase using this method (Figure 43b) [130]. In this study, the authors confirmed by HPLC analysis that the epimerization of the α‐position of each residue was < 1%. In 1997, Boger et al. [131] employed this *N*‐methylation protocol for the synthesis of the *N*‐methylcycloisodityrosine subunit found in deoxybouvardin or RA‐VII, that is, natural peptides with an interesting bicyclic structure and potent antitumor activity. Depending on the relative stereo configuration of the ring structure, some epimerization was observed in the *N*‐methylation step (Figure 43c). Very recently, Hitotsuyanagi achieved the *N*‐methylation of almost identical compounds using sodium or potassium hydroxide [132, 133]. Furthermore, this method has also been applied to the *N*‐methylation of cyclic peptide units in the synthesis of antibiotic arylomycins as reported by Romesberg [134] (Figure 43d).

**FIGURE 44:**
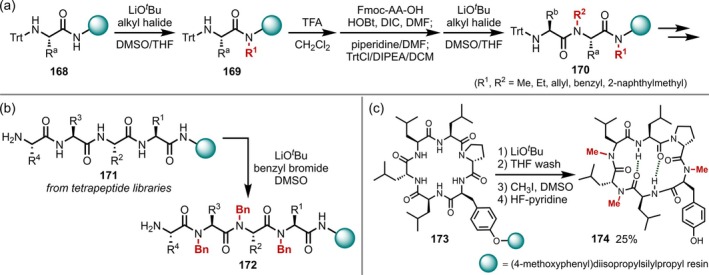
LiO^*t*^Bu‐mediated *N*‐alkylation of peptides on resins. (a) Sequential *N*‐alkylation during the SPPS. (b) “Libraries from libraries”. (c) Site‐selective *N*‐methylation of cyclic peptides on resins.

In 1996, Houghten also developed a method suitable for the sequential *N*‐alkylation in the solid phase by replacing sodium hydride with lithium *t*‐butoxide as a base (Figure 44a) [135]. Epimerization was not observed using this protocol, and a library comprising 57,500 peptide compounds was constructed by repeatedly elongating the peptide chains and introducing random alkyl groups (methyl, ethyl, allyl, benzyl, and 2‐naphthylmethyl) using this *N*‐alkylation protocol. In the same year, the same group also proposed the concept of “libraries from libraries,” in which they applied *N*‐alkylation to the peptide compound library in order to obtain a more diverse *N*‐alkylated peptide library (Figure 44b) [136]. In 2011, Jacobsen and Lokey applied these conditions to cyclic peptides supported on a resin and observed site‐selective *N*‐methylation derived from intramolecular hydrogen bonding, in contrast to the unselective complete *N*‐methylation observed in acyclic peptide chains, and successfully obtained compounds with high membrane permeability (Figure 44c) [137]. In 2018, Bode applied these conditions to peptide chains on a resin and conducted structure–activity relationship (SAR) studies using the obtained permethylated rhabdopeptides, which exhibited low activity against different protozoa, that is, the causative agents of several tropical diseases [138].

**FIGURE 45:**
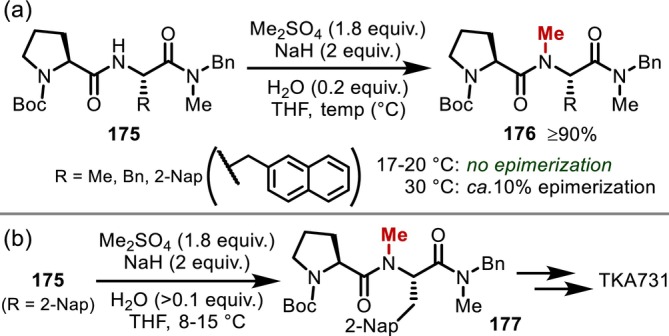
Large‐scale *N*‐methylation using NaH/Me_2_SO_4_ promoted by a small amount of H_2_O. (a) Reaction development. (b) Large‐scale synthesis of TKA731.

In 2003, process chemists at Novartis developed an efficient *N*‐methylation protocol employing dimethyl sulfate and sodium hydride in the presence of a small amount of water (Figure 45a) [139]. They explored the use of dimethyl sulfate instead of methyl iodide, which is undesirable in large‐scale synthesis due to its low boiling point and high toxicity, and discovered that the addition of a small amount of water to sodium hydride to generate highly reactive dry sodium hydroxide was the key to the successful *N*‐methylation of the peptides (**175**). They also investigated the relationship between the reaction temperature and α‐epimerization in detail. No epimerization was observed at temperatures < 20°C, whereas ~10% epimerization was observed when the temperature was raised to 30°C. The next year, they reported that they had successfully applied the protocol to the synthesis of TKA731, an NK1‐receptor antagonist, obtaining 23.4 kg of the final product in high purity (Figure 45b) [140].

**FIGURE 46:**
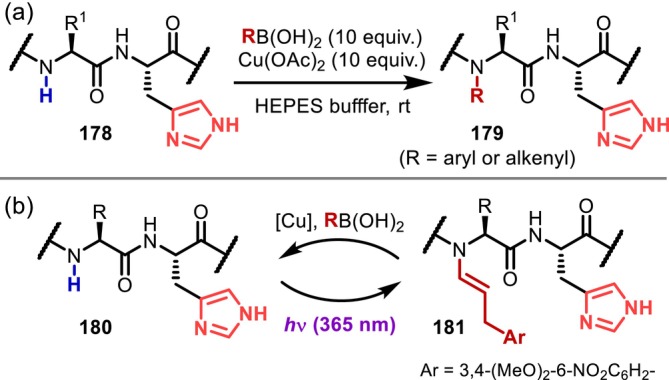
(a) His‐directed *N*‐arylation and alkenylation of peptides. (b) Reversible *N*‐alkenylation/dealkenylation.

The *N*‐alkylation approaches described above are essentially nonselective methods, which are advantageous for obtaining permethylated or randomly alkylated compounds. In contrast, in 2016, Ball reported the Cu‐catalyzed site‐selective *N*‐arylation and alkenylation of His‐containing peptides (**178**) (Figure 46a) [141]. Here, a Chan–Lam‐type coupling reaction using an aryl‐ or alkenyl‐boronic acid selectively proceeds on the amide nitrogen of the residue adjacent to the N‐terminal side of the His residue under mild conditions (at room temperature, in HEPES buffer), probably due to the strong direction via bidentate coordination with the His and amide in the main chain. The reaction proceeds particularly well with N‐terminal pyroglutamic acid (Glp), whereas it provides low yields at internal residues of peptides. In 2018, Ball discovered that the *N*‐alkenyl group introduced by their method can be easily removed by light irradiation (365 nm) to provide the original secondary amide (Figure 46b) [142]. Based on these findings, they demonstrated that a nine‐residue peptide could be temporarily inactivated against proteases. Subsequently, they also found that similar reactions proceed with Ni catalysts [143]. In addition, Gagnon reported in the context of their study on the *N*‐arylation of imidazole in His residues using triarylbismuth species that *N*‐arylation of the main‐chain amide proceeds, albeit in low yield, when the imidazole is protected with a trityl group [144]. However, this transformation is limited to amides between two glycine (Gly) residues [145].

**FIGURE 47:**
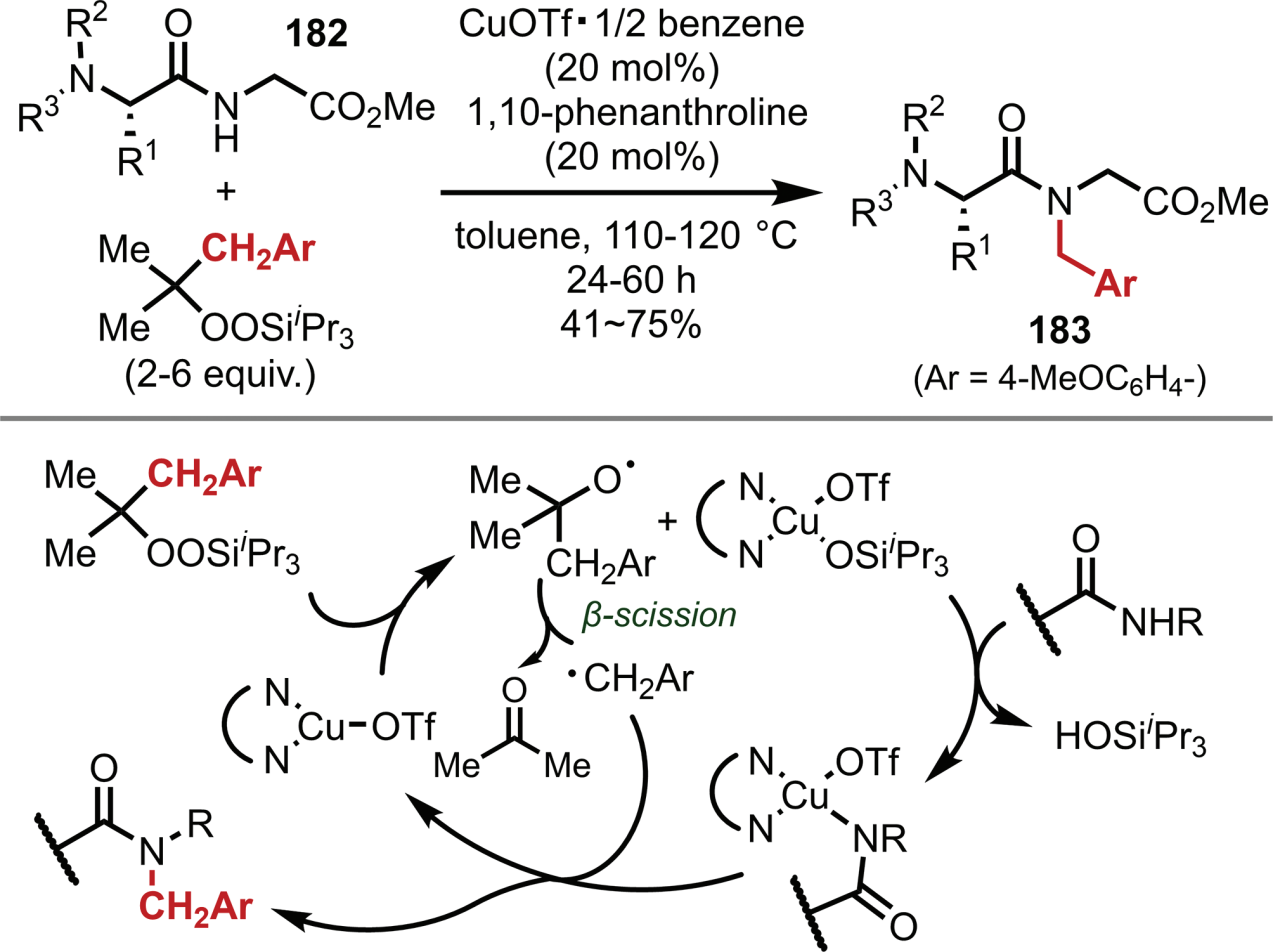
Radical‐mediated *N*‐4‐methoxybenzylation of amides in the peptide main chain promoted by a Cu catalyst.

In 2023, Maruoka reported the radical‐mediated *N*‐methoxybenzylation as an alternative approach to alkylation (Figure 47) [146]. The methoxybenzyl radical generated from alkylsilyl peroxide reacts with an amide/Cu complex, and subsequent reductive elimination yields the *N*‐4‐methoxybenzylated products (**183**). This reaction represents the only example of *N*‐alkylation in peptides via a mechanism involving radicals.

**FIGURE 48:**
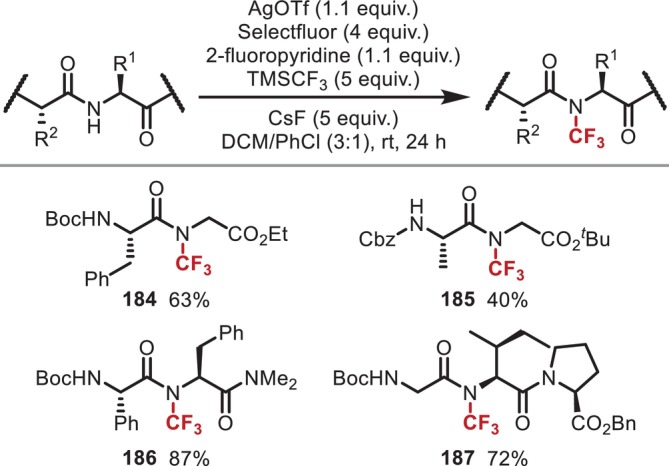
Ag‐mediated *N*‐trifluoromethylation of amides in the peptide main chain.

Another notable carbon‐substitution reaction on the amide of peptide main chains is *N*‐trifluoromethylation. In 2020, Fang and Li reported a trifluoromethylation promoted by Ag salts and Ruppert's reagent (TMSCF_3_) (Figure 48) [147]. In this study, the authors proposed that trivalent active Ag species, generated by Selectfluor oxidation of AgCF_3_ derived from TMSCF_3_, CsF, and AgOTf, are involved. CF_3_ groups are frequently incorporated into pharmaceutical compounds to improve the lipophilicity and metabolic stability of the latter; however, *N*‐trifluoromethylated amides are difficult to synthesize because the CF_3_–X moiety does not exhibit S_N_2 reactivity, and it is difficult to employ trifluoromethylamine in condensation reactions due to its low nucleophilicity. Thus, this method represents the first example of the synthesis of peptides with trifluoromethyl groups incorporated in the main‐chain amide.

### 3.2 Transformation of *N*‐Alkylated Peptides (Modification of Pro)

Another remarkable modification related to the nitrogen atom of the main‐chain amide is the oxidative transformation at the α‐position of the *N*‐alkyl group. The main target of this approach is the Pro residue, which is the only secondary amine among the 20 proteinogenic amino acids. Numerous transformations involving C–H functionalization at the 5‐position and dealkylative ring opening of the Pro residue triggered by hydrogen abstraction from α‐C–H bonds activated by hyperconjugation of the nitrogen have been reported.

**FIGURE 49:**
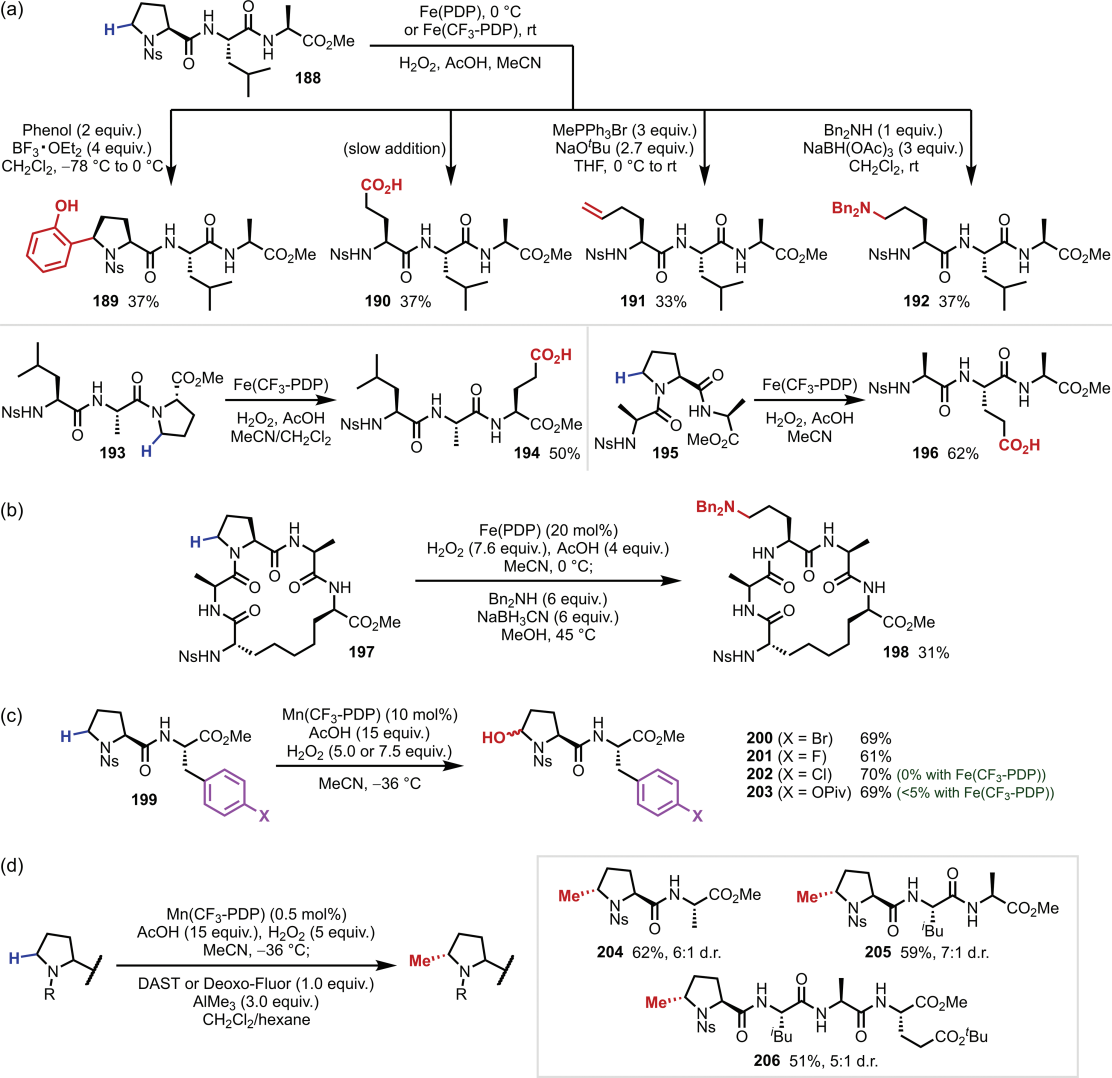
FePDP‐ and MnPDP‐catalyzed modification of Pro residues in oligopeptides. (a) Diversification of Pro‐containing tripeptides initiated by α‐C–H oxidation. (b) Ring‐opening amination of Pro residues in a cyclic peptide. (c) α‐C–H oxidation in the presence of Phe analogues. (d) Application to the α‐methylation of Pro residues.

In 2016, White reported the Fe(PDP)‐catalyzed α‐C–H oxidation of the Pro residue in peptides (Figure 49a) [22]. This reaction is applicable to oligopeptides regardless of whether the Pro residue is located at the N‐terminus, C‐terminus, or an internal position, and the α‐C–H oxidation product is obtained preferentially relative to the C(sp^3^)–H oxidation product of the aliphatic side chain as described in Section 2.1. Furthermore, the obtained hemiaminal can be converted to a variety of α‐arylated Pro residue derivatives via BF_3_‐catalyzed aromatic electrophilic substitution and can also be applied to Wittig reactions and reductive amination conditions to introduce unnatural side chains, even in the case of a macrocyclic peptide (**197**) (Figure 49b). In 2019, the same group reported that the catalyst Mn(CF_3_‐PDP) enables a similar transformation of the Pro residue even when a benzene ring, which would be oxidized in the Fe(PDP) system, is incorporated in the side chains (Figure 49c) [24]. In 2020, the same group reported the application of the Mn(CF_3_‐PDP) system to the α‐methylation of the Pro residue. Treatment of the α‐hydroxy Pro obtained by C–H hydroxylation with DAST or Deoxo‐Fluor and AlMe_3_ afforded methylated Pro‐containing peptides; however, this reaction was only applied to 4‐nitrobenzenesulfonyl (Ns)‐protected Pro at the N‐terminus (Figure 49d) [148].

**FIGURE 50:**
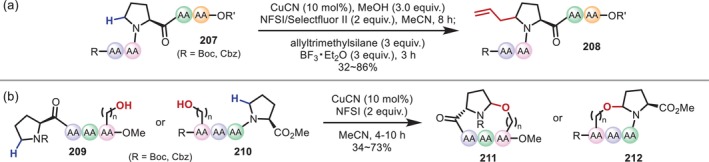
Cu‐catalyzed modification of Pro residues in oligopeptides. (a) α‐C–H allylation. (b) Intramolecular α‐C–H alkoxylation to provide cyclic peptides.

In 2023, Zhao reported a Cu‐catalyzed protocol as another example of a similar transformation involving aminal intermediates (Figure 50a) [149]. Here, allylated Pro and intramolecular cyclization products involving hydroxy groups were obtained from an oxidation promoted by a Cu catalyst and *N*‐fluorobenzenesulfonimide (NFSI) or selectfluor II followed by the nucleophilic addition of nucleophiles, even in a one‐pot protocol. Notably, the reaction proceeds even with internal Pro residues in oligopeptides (**207**), and in the cyclization reaction, the formation of macrocycles (**211**, **212**) with distant hydroxy groups was also achieved (Figure 50b).

**FIGURE 51:**
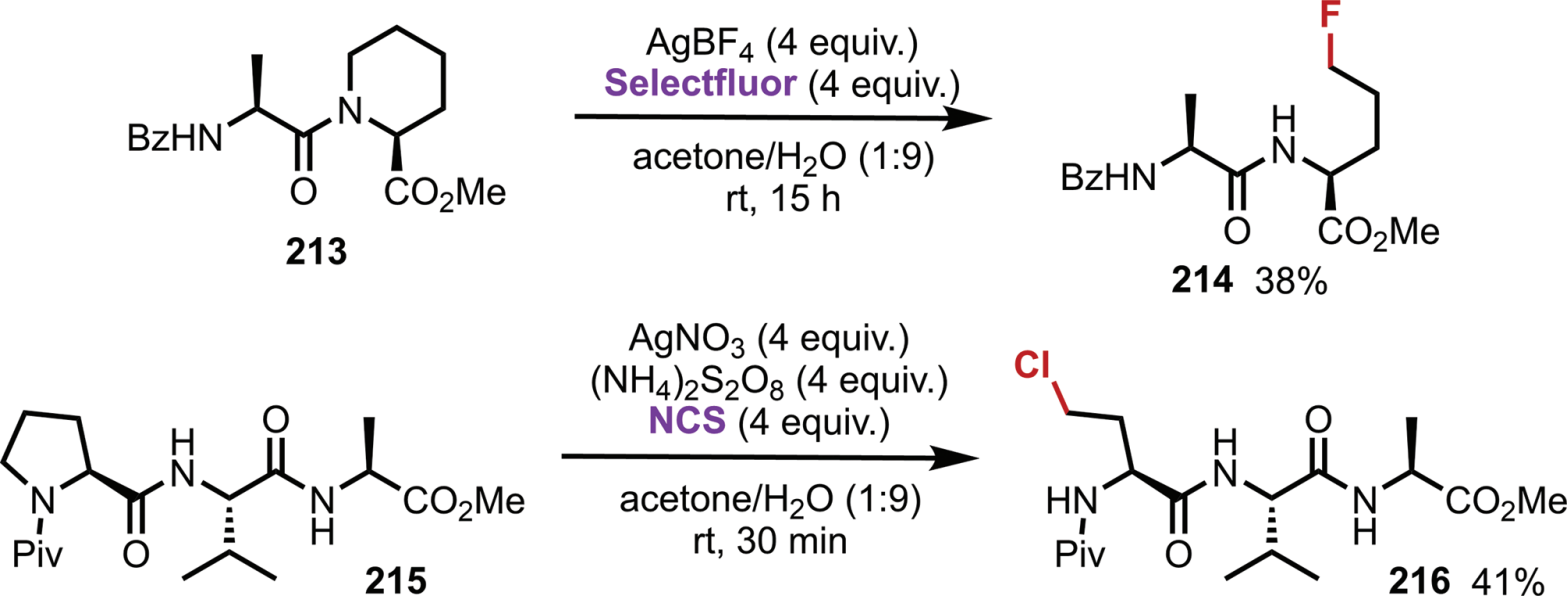
Ag‐mediated modification of Pip and Pro residues in oligopeptides.

In 2018, Sarpong reported the ring‐opening reactions of Pro and pipecolic acid (Pip) residues using Ag salts and oxidative halogenating agents (Figure 51) [150, 151]. The reaction conditions promoted the oxidative ring‐opening and the subsequent decarboxylative halogenation, providing the corresponding peptide products (**214** and **216**) with a fluorinated or chlorinated side chain. These conditions were applied to the transformation of internal Pip residues in a tripeptide.

Furthermore, other interesting reactions have been reported, such as azidations using hypervalent iodine by Waser [152], the α‐alkoxylation and methylation using electrode oxidation as well as the oxoammonium‐catalyzed α‐oxidation by Lin [153, 154], and the ring‐opening transformation of Pro residues triggered by Lewis acid–promoted single electron reduction of benzamide reported by Yamaguchi [155]. However, these reactions have generally only been performed with N‐terminal Pro residues protected with specific protecting groups.

### 3.3 Transformation of Amides to Other Functional Groups

**FIGURE 52:**
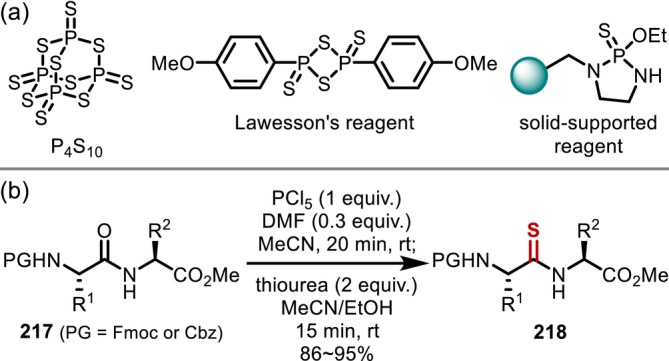
O–S exchange reactions in order to transform peptide amides into thioamides. (a) Phosphorus‐based O–S exchanging reagent. (b) O–S Exchange reaction of peptides through the electrophilic activation of amides.

In addition to the *N*‐alkylation reactions introduced in the previous section, the conversion of main‐chain amides to various functional groups by FGT has also been studied extensively. Such reactions essentially involve nucleophilic addition to an electrophilically activated amide; a representative example of this approach is O–S exchange reactions to convert amides into thioamides. Thioamides are isoelectronic sulfur‐containing analogues of amides and are often introduced into peptides as amide bioisosteres to endow peptides with higher resistance to metabolic hydrolysis [156]. Phosphorus sulfides such as diphosphorus pentasulfide [157] and Lawesson's reagent [158] can directly convert amides into thioamides. Since their application to peptides was first reported [159, 160], this approach has been applied to the synthesis of various peptides containing thioamides (Figure 52a). Later, polymer‐supported O–S exchange reagents were reported [161]; however, these invariably require high temperatures or long reaction times for reactions with peptides. In an alternative approach, Sureshbabu reported in 2016 stepwise reactions using phosphorus pentachloride, which can produce the desired thioamides (**218**) at room temperature within a short reaction time (Figure 52b) [162]. Nevertheless, these reactions are fundamentally limited to dipeptides, and site‐selectivity becomes a serious concern even if they can be applied to longer peptide chains. Therefore, in the actual synthesis of thioamide‐containing peptides, peptide elongation using thioacylating agents [163], which are also applicable to Fmoc SPPS [164, 165], is primarily employed.

**FIGURE 53:**
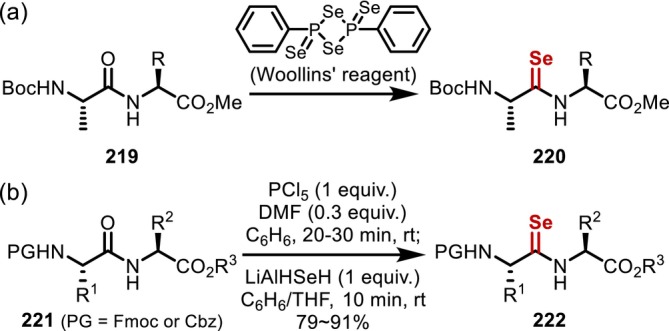
Synthesis of selenoxopeptides through O–Se‐exchange reactions; (a) with Woollins' reagent; (b) with PCl_5_, DMF, and LiAlHSeH.

In addition, there have also been a few reports of selenoamide synthesis based on an O–Se exchange. In 2010, Fischer reported the synthesis of selenoxo dipeptides (**220**) from the corresponding native peptides (**219**) using Woollins' [166] reagent, together with an interesting reversible interconversion between *cis*‐ and *trans*‐ isomers upon exposure to heat or UV irradiation (Figure 53a) [167]. In 2012, Sureshbabu reported a mild protocol for the O–Se exchange of peptides; stepwise activation–selenolation promoted by phosphorus pentachloride and Ishihara reagent (LiAlHSeH) proceeds at room temperature within a short reaction time (Figure 53b) [168].

**FIGURE 54:**
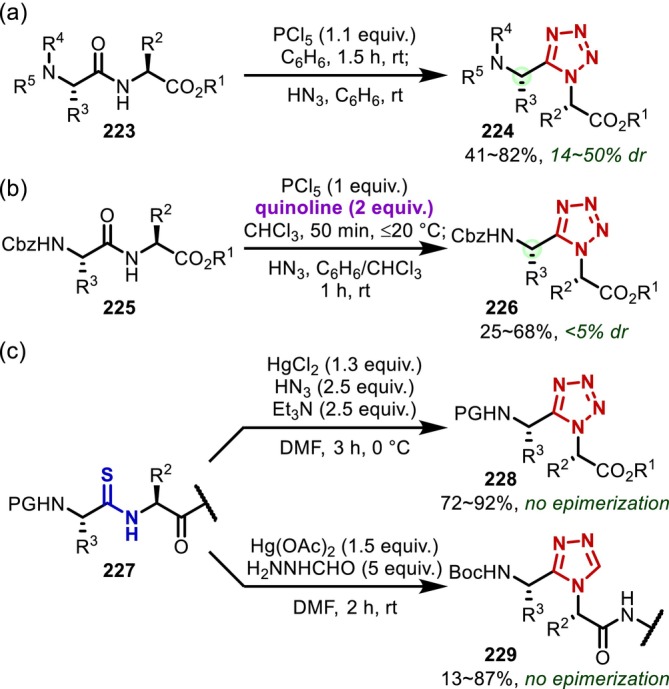
Synthesis of heterocycle‐containing peptidomimetics. (a) Synthesis of tetrazole with HN_3_. (b) Epimerization‐free conditions for the formation of tetrazole. (c) Hg‐Mediated conversion of thioamide to tetrazole and triazole.

Another main‐chain amide modification based on a nucleophilic attack on activated amides is the conversion to heterocycles. In 1987, Yu and Johnson [169] found that main‐chain amides can be converted into tetrazoles, that is, *cis*‐amide isosteres, by treating dipeptides (**223**) with phosphorous pentachloride and azide anions, albeit that significant epimerization of the N‐terminal side of the reacting amide was observed (Figure 54a). The following year, Marshall found that adding quinoline during the formation of the imidoyl chloride intermediate significantly suppresses epimerization [170]. Later, it was shown that in addition to quinoline, a series of pyridine derivatives also suppress epimerization (Figure 54b) [171]. An alternative approach to obtain tetrazole‐containing peptides involves a thioamide intermediate; this method has also been applied to the conversion to triazoles (**229**) using formohydrazide as a nucleophile, although a highly toxic mercury Lewis acid is often required to activate the thioamide intermediates (**227**) (Figure 54c) [172, 173].

## 4 Modification of the α‐Position in Peptide Main Chains (Modification of Gly Residues)

The chemical modification of the α‐position of the peptide main chain is another attractive approach to introduce unnatural amino acid structures. The Gly residue, as the only α‐amino acid without substituents at the α‐position, is suitable for such modifications due to its good steric accessibility. Accordingly, the introduction of substituents at the α‐position of the Gly residue in peptides has been studied extensively. Conversely, this also implies that the conversion of residues other than Gly is difficult, considering the site‐selectivity of the transformation, and indeed, modifications targeting α‐substituted amino acid residues remain so far limited to amino acid monomers.

**FIGURE 55:**
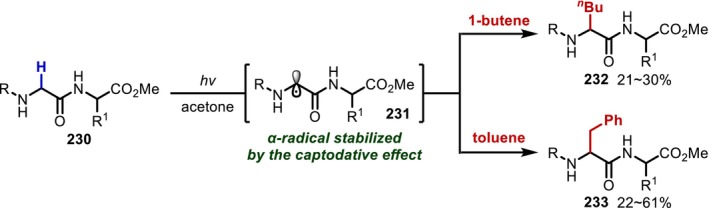
Excited ketone‐mediated α‐alkylation of Gly residues in peptides.

In 1969, Elad and Sperling [174] reported a UV‐mediated alkylation reaction of the Gly residue in peptides (**230**) triggered by HAT at the α‐position induced by excited acetone (Figure 55). The resulting α‐radicals (**231**) are captodative radicals [175] strongly stabilized by both the adjacent electron‐donating nitrogen substituent and the electron‐withdrawing carbonyl group. Not only can they be trapped by 1‐butene to yield Nle residues, but they can also participate in radical–radical coupling with benzyl radicals generated from toluene to yield Phe residues in moderate yield. Even though the d/l ratio of the alkylated residue is essentially 1:1, the same authors applied this method in 1971 to polypeptides, which revealed that polypeptides with repetitive sequences show greater asymmetric induction [176]. In 1973, the same group reported that a similar transformation could be achieved under visible light irradiation using α‐diketone as a HAT mediator [177].

**FIGURE 56:**
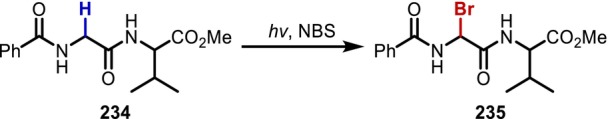
NBS‐mediated α‐bromination of Gly residues in peptides.

In 1986, Easton and Hay [178] reported that α‐bromination of the Gly residue in dipeptides (**234**) proceeds with NBS under heating and UV‐irradiation conditions (Figure 56). Here, the α‐position of the Gly residue is the most reactive among those of Gly, Ala, and Val, which was attributed to the stability of the generated captodative radical; that is, only Gly has no steric repulsion between the alkyl group of the side chain and the carbonyl group at the N‐terminus. It was also demonstrated that the prepared α‐brominated peptides (**235**) can be converted into α‐deuterated or methoxy derivatives, Asp residues, and allylglycine, respectively [179]. Relatedly, the same group also found that the α‐radical of phthaloyl‐protected Gly is unfavorable and that the α‐bromination occurs selectively at the C‐terminal Gly in Gly‐Gly dipeptides with phthaloyl protection on the N‐terminus [180].

**FIGURE 57:**
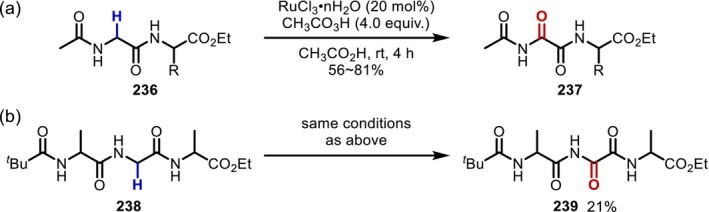
Ru‐catalyzed α‐oxidation of Gly residues in peptides. (a) Oxidation of a dipeptide. (b) Oxidation of an internal Gly residue in a tripeptide.

In 2000, Murahashi et al. [181] reported a Ru‐catalyzed Gly‐selective α‐oxidation to provide carbonyl derivatives (**237**) (Figure 57a). A radical mechanism involving C–H abstraction by the Ru‐oxo species generated by peracetic acid was proposed. Although the yield was not high, this is one of the rare examples in which α‐modification of an internal Gly residue in oligopeptides (**238**) has been successfully achieved (to the best of our knowledge, there are only three examples of the α‐modification of an internal Gly residue in oligopeptides) (Figure 57b).

**FIGURE 58:**
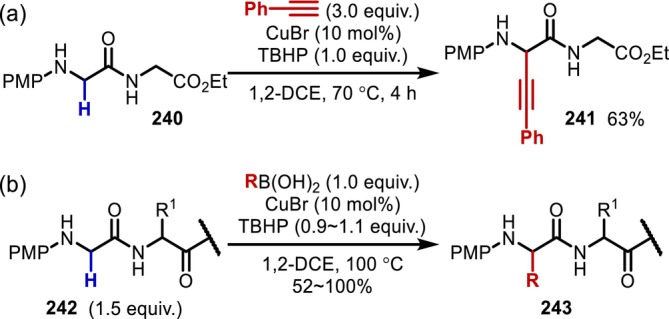
α‐modification of Gly residues in peptides promoted by a Cu(II)/TBHP system. (a) Alkynylation with an alkyne. (b) Alkylation with a boronic acid.

In 2008, Zhao and Li [182] reported the α‐alkynylation of Gly residues using a Cu catalyst/*t*‐butylhydroperoxide (TBHP) system (Figure 58a). The authors argued that Cu(II) acts as an oxidizing catalyst on the N‐terminal Gly substituted with a methoxyphenyl group to form an imine intermediate, which then reacts with a terminal alkyne assisted by the Cu species. In 2019, coupling with a wide variety of boronic acids was reported, suggesting that the secondary amide on the C‐terminal side of the Gly residue is important for the activation of boronic acids (Figure 58b) [183].

**FIGURE 59:**
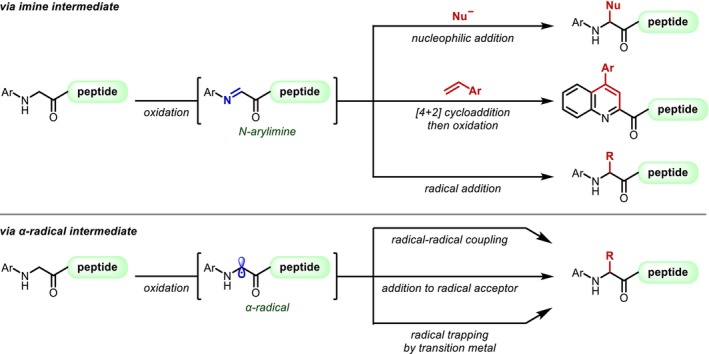
Summary of the α‐functionalization of *N*‐aryl substituted Gly residues on the N‐terminus.

Since these pioneering reports by Zhao and Li [182], the α‐modification of N‐terminal Gly residues with *N*‐aryl substituents has been studied extensively, with over 50 papers published by 2024 (Figure 59). Although we will not introduce all of these considering the focus of this review on the transformation of inert peptides, all these reactions are limited to *N*‐aryl‐substituted Gly N‐termini, which are more reactive in oxidative transformations than acyl‐ or carbamoyl‐substituted ones due to their redox potential and the electron‐richness of the α‐C–H bonds.

These reactions can be broadly divided into two categories based on the reaction pathway of the bond‐forming step at the α‐position, that is, reaction through an imine or reaction through an α‐radical intermediate. The reported reactions involving imines are mainly nucleophilic additions of nucleophiles [184, 185, 186, 187, 188, 189, 190], similar to the reactions reported by Li [182, 183], [4 + 2] cycloadditions [191, 192, 193, 194, 195], or radical additions [196]. Among the reactions involving α‐radicals, numerous reports based on radical–radical coupling [197, 198, 199, 200] using the stability of captodative α‐radicals have been published, together with a few reports on reactions involving radical acceptors [201] or trapping by a transition metal [202]. Additionally, the mechanisms of the oxidation step are primarily hydrogen abstraction [186, 187, 191, 196, 202] and deprotonation at the α‐position following single electron oxidation, which is particularly facile with arylamines [184, 188, 190, 192, 193, 194, 195, 198, 199, 201], and the mediators inducing the oxidation are mainly transition metal catalysts [186, 187, 193, 194, 197] and photoredox catalysts [184, 188, 190, 192, 195, 198, 201].

**FIGURE 60:**
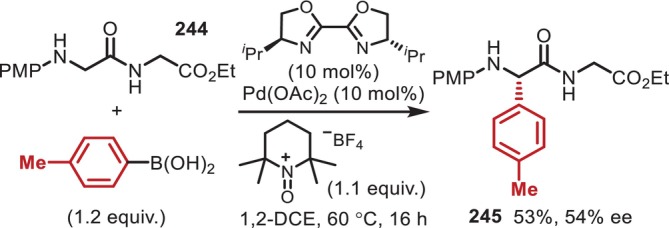
Asymmetric α‐modification of Gly‐Gly dipeptides.

Although these reactions have the inherent disadvantage of providing mixtures of stereoisomers at the α‐carbon, Yang reported in 2015 a Pd‐catalyzed asymmetric arylation of a Gly‐Gly dipeptide (**244**) using a chiral bisoxazoline ligand, albeit that the stereoselectivity was low (Figure 60) [203]. In 2020, Zhang reported a highly stereoselective asymmetric Gly‐Gly alkylation promoted by Cu/chiral amine co‐photocatalysis [204]. In 2021, Chen applied the α‐modification of the Gly residue to the synthesis of macrocyclic peptides [205], whereas Young and Proulx [206] reported in 2022 that the α‐alkylation with alkylboronic acids also proceeds on resins.

**FIGURE 61:**
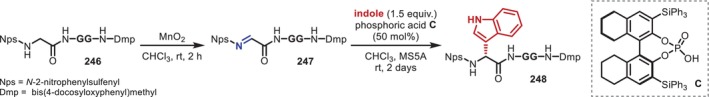
Chiral phosphoric acid‐catalyzed stereoselective α‐indolylation of Nps‐protected Gly residues in oligopeptides.

As an interesting reaction other than the modification of the Gly residue activated by the *N*‐aryl group discussed above, Otaka reported in 2018 a chiral phosphoric acid‐catalyzed stereoselective addition of indole to a Gly‐derived imine protected with an *N*‐2‐nitrophenylsulfenyl (Nps) group (Figure 61) [207]. Gly residues were successfully converted to imines with the assistance of MnO_2_, which then react with indole to afford the desired adducts. It was also demonstrated that elongation of the peptide chain is possible after Nps deprotection, albeit that the stereoselectivity is moderate.

**FIGURE 62:**
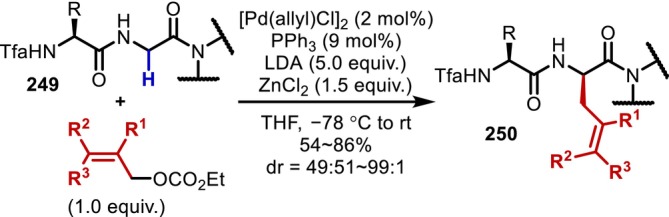
LDA‐mediated diastereoselective α‐allylation of Gly residues in oligopeptides.

In 2012, Kazmaier reported a completely different approach from those described above based on α‐oxidation [208]. Here, α‐allylation is achieved via the selective deprotonation of Gly residues in peptides (**249**) using lithium diisopropylamide (LDA) and a subsequent diastereoselective Pd‐catalyzed allylic substitution (Figure 62). Later, the same group also reported branched‐chain allylation using Ru catalysts via a similar approach. These reactions are among the few that proceed at internal Gly residues of oligopeptides [209].

## 5 *N*‐Chloropeptide Strategy for the Conversion of Inert Aliphatic Peptides

Up to this point, various cutting‐edge approaches for converting “chemically inert peptides” using the latest advances in synthetic organic chemistry have been presented. However, these reactions usually require harsh conditions such as strong bases or heating and/or the preinstallation of specific motifs such as directing groups or activating groups in order to enable the transformation of unreactive molecules. Thus, significant limitations remain in terms of applicable substrates and situations.

**FIGURE 63:**
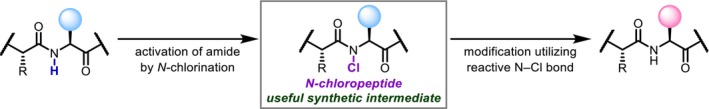
*N*‐Chloropeptide strategy for the chemical modification of peptides.

In this context, we have developed an “*N*‐chloropeptide strategy” involving the *N*‐chlorination of amides in the peptide main chain. This represents an approach for the chemical modification of peptide compounds that does not depend on the native reactive functional groups of the side chains or preinstalled activating groups (Figure 63) [210, 211]. As discussed in Section 3.1, reactions for the introduction of *N*‐substituents, such as *N*‐alkylation, on secondary amides typically require strong bases; in contrast, *N*‐chlorination proceeds smoothly with simple amide substrates [212, 213]. We envisioned that the amide groups of the peptide backbone could also be smoothly chlorinated under similar conditions and that the resulting *N*‐chlorinated peptides could serve as an activated synthetic intermediate for further chemical modification of the neighboring residues, taking advantage of the high reactivity of N–Cl bonds.

**FIGURE 64:**
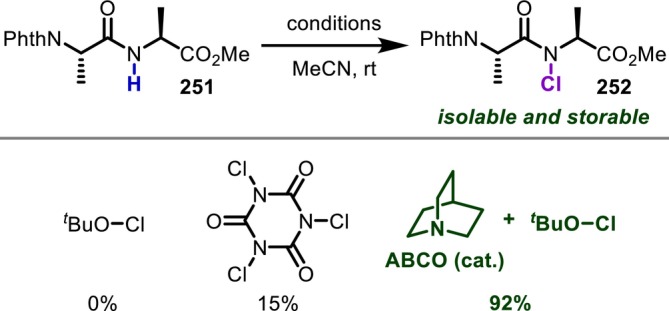
Optimization of the *N*‐chlorination reaction of main‐chain amides in peptides.

We first investigated the *N*‐chlorination of amides in an Ala‐Ala dipeptide (**251**) with phthaloyl protection on the N‐terminus. The reactions were ineffective for peptides due to the low reactivity of the peptide‐backbone amide, even though *t*‐butyl hypochlorite (^*t*^BuOCl) and trichloroisocyanuric acid (TCCA), which had been reported to be effective with simple substrates [212, 213], were used (Figure 64) [210]. Following a detailed screening of the reaction conditions, the addition of a catalytic amount of 1‐azabicyclo[2.2.2]octane (ABCO, quinuclidine) to ^*t*^BuOCl was found to drastically accelerate the *N*‐chlorination, and the reaction proceeded even in aqueous solvents. The obtained *N*‐chloropeptide (**252**) was easily isolated via column chromatography over silica gel and characterized using X‐ray diffraction analysis; moreover, it was found to be shelf‐stable for at least several months.

**FIGURE 65:**
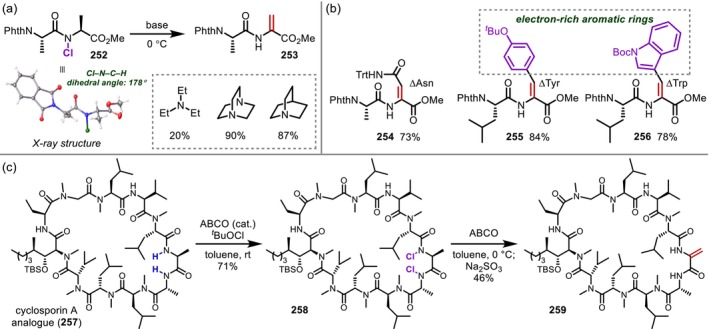
Dehydrogenation of peptides through *N*‐chloropeptides and its application to the late‐stage modification of macrocyclic peptides. (a) Investigation on β‐elimination/isomerization. (b) Substrate scope for ΔAA formation. (c) Application to the late‐stage dehydrogenation of macrocyclic peptides.

Subsequently, we also explored the derivatization of peptides through *N*‐chloropeptide intermediates prepared using the conditions outlined above. First, we discovered that treatment with a bicyclic tertiary amine such as ABCO promotes β‐elimination and subsequent isomerization of the double bond to provide the dehydroamino acid (ΔAA) structure, an abnormal amino acid motif. The X‐ray diffraction structure of the *N*‐chloropeptide shows a conformation ideal for β‐elimination, in which the chlorine atom and the hydrogen atom at the α‐position of Ala are in an almost antiperiplanar relationship (Figure 65a) [210]. This reaction can be performed as a one‐pot process combining the *N*‐chlorination step and successfully converted various amino acid side chains into the corresponding ΔAA structures in good yield. Notably, despite the use of strong oxidative conditions during the *N*‐chlorination step, the reaction proceeds without problems, even in the presence of electron‐rich aromatic rings in, for example, Tyr and Trp residues (Figure 65b). Furthermore, the late‐stage installation of an ΔAA motif into a macrocyclic peptide was achieved, showcasing the potential of this method. Cyclosporin A, a calcineurin inhibitor used as an immunosuppressant medication, is a macrocyclic undecapeptide consisting mainly of aliphatic side chains. Its analogue **257** was dichlorinated under the aforementioned conditions, and subsequent treatment with ABCO provided the mono‐dehydrogenated product (**259**) as a single regioisomer in moderate yield (Figure 65c). This is the first example of the late‐stage introduction of an ΔAA motif into macrocyclic peptides, which demonstrates that the *N*‐chloropeptide strategy represents a powerful approach to modify chemically inert aliphatic peptides.

**FIGURE 66:**
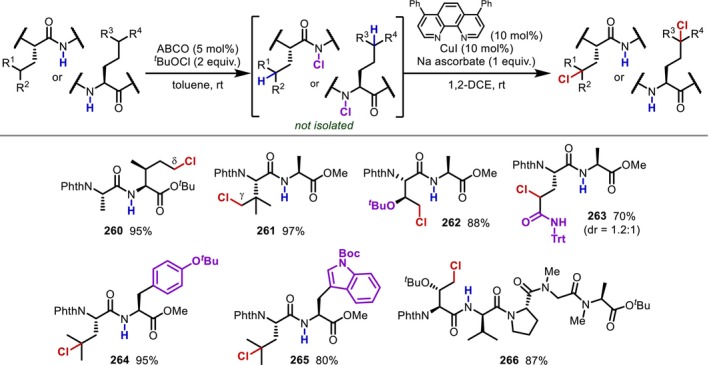
Side‐chain chlorination of γ‐ and δ‐C–H bonds in peptides through an intramolecular HAT process.

*N*‐chloropeptides can also be used for the radical‐mediated C–H functionalization of peptide side chains. As discussed in Section 2.1, the C–H bonds in peptide side chains are generally poorly reactive in intermolecular HAT processes; on the other hand, they react very well with the intramolecular amidyl radical generated from *N*‐chloroamides with the assistance of Cu(I)/1,10‐phenanthroline catalysis, enabling the chlorination of a wide range of γ‐ and δ‐C–H bonds, including primary C–H bonds that are typically unreactive in radical reactions (Figure 66) [211]. This method allowed the synthesis of the side‐chain‐chlorinated peptide fragment of the natural product aquimarin A, which had been difficult to synthesize using previous methods, and an estimation of the stereochemical configuration of the side‐chain structure by comparing the spectral data of prepared compounds. Furthermore, it also enabled the introduction of a wide range of substituents into the side chain via subsequent conversion of the C–Cl bonds of the product.

The “*N*‐chloropeptide strategy” has already been proven useful in our group for a wide range of applications, such as the modification of main‐chain amides, and further details will be published elsewhere in the near future.

## 6 Summary and Outlook

This review discusses pioneering approaches for the chemical modification of peptides based on recent advances in synthetic organic chemistry. The chemical modification of peptides and proteins through conventional functional group transformation of reactive residues is widely performed as a relatively reliable approach for various applications. However, targetable functional groups are not always present at the desired position in peptides, and thus, the development of fundamentally different chemical modification approaches that can be employed even in the absence of suitable reactive sites in complex molecules is undoubtedly one of the most challenging but worthwhile research topics for contemporary organic chemists. Through the collective efforts of many researchers, interesting approaches using various reaction mechanisms and catalysts, as presented in this review, have started to emerge. Although these approaches are still mainly focused on short oligopeptides as reaction substrates due to the extraordinary difficulty of modifying even bigger chemically inert complex molecules, we are convinced that exciting breakthroughs will be achieved in the near future through further progress and expansion of these efforts, leading to innovative approaches that will eventually make the currently “impossible” possible.

## Funding

This study was supported by the Japan Society for the Promotion of Science (JP22H02743 and JP23K24006) (T.N.). The authors also gratefully acknowledge JST SPRING for Grant JPMJSP2110 (H.S.).

## Conflicts of Interest

The authors declare no conflicts of interest.

## Data Availability

Data sharing is not applicable to this article, as no datasets were generated or analyzed.
